# A hybrid system for detecting semiconductor wafer defects using modified MobileNet with multi-head attention

**DOI:** 10.1371/journal.pone.0346595

**Published:** 2026-04-08

**Authors:** Sharith Dhar, Fahmid Al Farid, Md Saiful Islam, Jia Uddin, Sarina Mansor

**Affiliations:** 1 Department of Electronics and Telecommunication Engineering, Chittagong University of Engineering & Technology, Chittagong, Bangladesh; 2 Centre for Image and Vision Computing (CIVC), COE for Artificial Intelligence, Faculty of Artificial Intelligence and Engineering (FAIE), Multimedia University, Cyberjaya, Selangor, Malaysia; 3 AI and Big Data Department, Woosong University, Daejeon, South Korea; Hohai University, CHINA

## Abstract

Identifying defective semiconductor wafers is a crucial and complicated aspect of the manufacturing process. Developed systems often confront difficulties in capturing intricate defect patterns, as well as the long-range relationships between defects and small defects within wafers. To address these issues, this paper proposes a hybrid system that combines a modified MobileNet structure with an error-correcting output code (ECOC)- based support vector machine (SVM) classifier. In the proposed method, for feature extraction purposes, the MobileNet architecture is modified 1) by using the Swish function instead of the ReLU function in the depth-wise separable convolution block of the MobileNet structure for detecting complex defective patterns and 2) by incorporating the multi-head attention mechanism in the MobileNet architecture for capturing long-range dependencies on defective wafers. To solve the dataset class imbalance issue, the ECOC-SVM classifier is used in the proposed method. For enhancing the visibility of minor defects, the histogram equalization technique is applied to wafer images. The real-world semiconductor wafer dataset WM-811K is used in this study. This paper presents an ablation study to validate the necessity of each modification to the proposed system. The proposed system achieved a superior testing accuracy of 98.55% and better average values of AUC (99.74%), recall (93.34%), precision (95.64%), and F1-score (94.42%) compared with the original version of MobileNet. Also, Comparative analyses of the proposed system with other developed systems and state-of-the-art models are given in this paper.

## 1 Introduction

Despite Significant advancements in semiconductor fabrication industries, detecting and categorizing defects in wafers remains critical due to the difficulty of identifying macro-level defects in wafers and analyzing intricate defect patterns. Also, identifying long-range dependencies in wafer surfaces is a major concern in the semiconductor manufacturing process.

These challenges hinder the ability to ensure the quality and reliability of semiconductor devices. This paper addresses these challenges by proposing a modified MobileNet with a multi-head attention-based hybrid system that uses an ECOC-based SVM classifier for classifying wafer defects. Also, the proposed hybrid method utilizes histogram equalization to improve the visibility of small defects. The proposed technique improves semiconductor manufacturing efficiency and reliability by increasing defective wafer detection capability.

There are several types of wafer defect patterns. Among them, ‘Local’ defect patterns exhibit small defects. The ‘Donut’, ‘Edge-ring’, and ‘Scratch’ defect classes are complex defective patterns, and to identify these types of patterns, the defect detection model needs to recognize the long spatial relationship of defects in wafers. Classifying defects in semiconductor wafer manufacturing is crucial for early identification of potential defects, facilitating prompt corrective measures, and reducing expensive waste. The process relies significantly on manual inspection or basic automated systems. Nonetheless, the emergence of artificial intelligence (AI), machine learning (ML), and deep learning (DL) techniques has significantly enhanced the capability of identifying wafer defects and reduced the dependency on human expertise. A wide range of research has been carried out to identify defect patterns in wafers.

Traditional ML techniques detect patterns and understand structured data. Linear regression, logistic regression, SVMs, decision trees, k-nearest neighbors (KNN), and k-means clustering are important methods. SVM is the most powerful and widely used conventional machine learning approach for classification purposes. This technique finds the best hyperplane to separate data points from different classes. The purpose is to optimize the margin, defined as the distance separating the hyperplane from the nearest data points of each class. Nearest points are called support vectors because they define the hyperplane [1]. Ma et al. [2] analyze several aspects of SVM applications, including imbalanced dataset issues. Cervantes et al. [3] provide a summary of the challenges associated with SVM implementation in engineering fields and the real-world problem-solving capabilities of SVM. The KNN is a conventional ML algorithm frequently employed for classification and regression tasks. KNN is an instance-based learning algorithm that does not assume any underlying distribution. It classifies data points by examining the classes of their closest neighbors. The parameter k denotes the number of nearest data points considered during the classification decision-making process [4]. S. Zhang [5] investigates the challenges of using KNN algorithms with unbalanced datasets and suggests classification rules for solving imbalanced data issues. Sehih et al. [6] developed a machine learning-based system that identifies white pixel defects in the wafer. Mohiuddin et al. [7] suggested a wavelet entropy-based optimization approach for bearing fault classification, employing four ML algorithms, including SVM and KNN, for classification purposes. Hoque et al. [8] developed a crop yield prediction system that used three ML techniques.

Deep learning, especially Convolutional Neural Networks (CNNs), has transformed defect detection by automating feature extraction and learning directly from unprocessed data, including images or sensor inputs. CNNs are proficient at learning spatial hierarchies, rendering them very effective for image-based defect detection in semiconductor production. Zheng et al. [9] designed a deep CNN model that reduces temporal and spatial complexity without requiring large-scale raw wafer map data. In this model, the random sampling method is applied. However, this model struggles to detect small defects in wafers. A small-scale defect identification system has been suggested by Byun et al. [10] using the rotated defects (RoD) transform technique and a modified version of median filtering. Misra et al. [11] used soft voting to improve performance after acquiring the deep ensemble characteristics for defective wafer identification. The attention mechanism and cosine normalization-based wafer map analysis method developed by Xu et al. [12] effectively addresses the problem of dataset imbalance. But this model faces problems in detecting complex patterns and small defects in wafers. E. Sin and C. D. Yoo [13] present a wafer defect classification method that requires no high-performance hardware. Chen et al. [14] designed a dual-source DCNN architecture to detect defect patterns of wafers. However, this model encounters challenges in finding long-range defect linkage in defective wafers. A contactless inspection system constructed by Jeon et al. [15] used thermal images and CNNs to detect printed circuit board assembly (PCBA) defects. The multi-scale residual dilated convolution attention mechanism network super-resolution reconstruction algorithm proposed by X. Sun et al. [16] for defect wafer detection. An automatic visual defect identification system developed by Limam et al. [17] uses different types of CNN architectures. M. Shahroz et al. [18] proposed a hierarchical attention module-based CNN model to detect hotspots in semiconductor wafer manufacturing. Deng et al. [19] developed a light-weight neural network-based system for the identification of mixed-type wafer defects. Hou et al. [20] developed a mixed defect type detection and classification system that constructs an improved version of the DCNN architecture, defined as the multi-path DCNN. Ma et al. [21] developed a method that identifies tiny defects using a spatial attention block and a residual architecture. López et al. [22] proposed a semiconductor wafer defect detection method that incorporated a computer vision technique with a lightweight SqueezeNet. A mixed defect wafer classification method proposed by J. Shim and S. Kang [23] uses the single defect wafer training dataset. The mix-up, random rotation, and noise filtering are applied in this suggested technique to create synthetic wafer maps from the training dataset. Hossain et al. [24] developed a depth-wise separable convolution-based modified and reduced MobileNet model that recognized plant leaf diseases.

Hybrid methods integrate traditional ML and DL techniques, seeking to capitalize on the advantages inherent in both approaches. Hybrid systems frequently combine traditional ML algorithms with DL models to enhance performance. Chen et al. [25] developed a hybrid system for the defective wafer map patterns recognition that used the improved version of the convolution block attention module (CBAM) with the deep CNN architecture for feature extraction and an ECOC-based SVM for classification purposes. However, this model has a large number of parameters. Schlosser et al. [26] proposed a hybrid semiconductor wafer fault inspection system that integrates classical computer vision techniques for fault localization and deep neural networks (DNNs) for classification. This system focuses on detecting very tiny defects in wafers. For satellite image classification, Tumpa et al. [27] proposed an SVM-based lightweight parallel CNN model that used the EuroSAT and RSI-CB256 datasets.

However, the semiconductor wafer detection methods developed above have some limitations. Some of these models struggle to identify both complex defect patterns and spatial dependencies among defects on the defective wafer, such as the ‘Edge-ring’, ’Donut’, and ‘Scratch’ defect classes. Few models fail to correctly identify ‘Local’ defects due to their inability to detect tiny defective regions in wafers. These disadvantages lead to the problem of erroneous defective wafer classifications. Several state-of-the-art models, such as MobileNet V1 [28], MobileNet V2 [29], ResNet-18 [30], and ResNet-50 [30], are used for detecting defect wafers, but these are unable to solve the existing problems mentioned above.

To overcome these limitations, we propose a hybrid system consisting of a modified MobileNet with multi-head attention and an ECOC-based SVM classifier. The main contributions of this paper are given below:

i This paper modifies the depth-wise separable convolution (DSC) block of the original version of MobileNet by using the swish function in place of the ReLU function to find features in complicated patterns.
ii This paper incorporates the multi-head attention mechanism in a modified MobileNet architecture to effectively capture long-range dependencies in defective wafers.
iii This study employs the ECOC-based SVM classifier within the proposed hybrid system to tackle class imbalance challenges alongside the classification task.
iv This paper applies histogram equalization to the wafer map dataset to improve the visibility of minor defects within wafers.
v This paper utilizes the well-recognized semiconductor wafer data set WM-811K to validate the effectiveness of the proposed system.

The remainder of this paper is organized by following major sections: Section 2 presents the problem statement, Section 3 explains the methodology, Section 4 provides an analysis of the results, and finally, conclusions are given in Section 5.

## 2 Problem statement

Identifying complex defective wafer patterns and detecting long-distance dependencies of defects on wafers are challenging tasks for traditional CNN models [10,14] due to their limited receptive field and lack of non-local operations. These models [10,14] fail to effectively detect the ‘Edge-ring’, ‘Donut’, and ‘Scratch’ defect classes.

**Fig 1:**
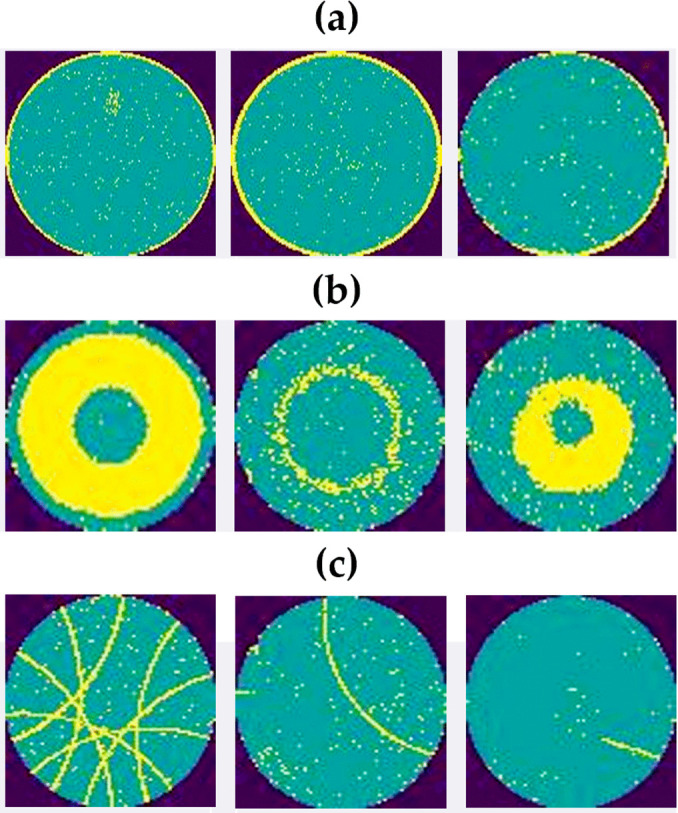
Complex defective wafer patterns. Notes: (a) Edge-ring, (b) Donut, (c) Scratch.

Among state-of-the-art models, the MobileNet structure [28] utilizes depth-wise separable convolutions (DSC), which significantly reduce computational complexity and the number of parameters. Despite these advantages, the MobileNet architecture [28] faces problems in identifying complex patterns in wafers. Fig 1 depicts ‘Edge-ring’, ‘Donut’, and ‘Scratch’ defect patterns. The ‘Edge-ring’ defect is complex because of its natural circular form, non-local spatial relationships, geometric invariance, and incomplete patterns. The ‘Donut’ defects involve a ring-shaped defect pattern where a central region is defect-free, surrounded by a ring of defects. Its non-local geographical distribution makes this a complicated pattern. The ‘Scratch’ defects manifest as elongated, narrow lines or curves that run the wafer in diagonal, radial, or spiral patterns. This is also a complex defect pattern because these patterns lack consistent orientation.

**Fig 2:**
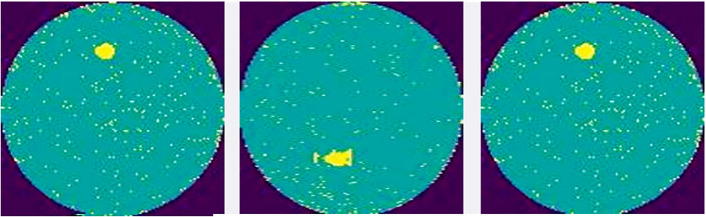
Local defective wafer patterns.

The DSC block of the MobileNet structure [28] uses the ‘ReLU’ function, which makes the MobileNet unable to detect these types of complex patterns. Additionally, the original MobileNet version [28] faces challenges in identifying the relationship of long-distance defects in the ‘Edge-ring’, ‘Donut’, and ‘Scratch’ type patterns in defective wafers, as shown in Fig 1. Identifying these patterns, the defect detection model needs to focus on relationships of defects situated in distant parts of the wafers to identify the continuation of defects; for this task, the attention mechanism is required, which is absent in the original MobileNet structure [28]. Detecting small defects such as ‘Local’ defect pattern is also a difficult task for existing models [9,10,12]. Fig 2 depicts the ‘Local’ defect patterns, which have tiny clusters of defects that are only found in a single region of the wafer. Developed models fail to recognize this small defective region in wafers. To enhance the visibility of the features of tiny defective wafers, an image pre-processing technique is required.

To overcome these issues, this paper proposes a multi-head attention mechanism-based modified MobileNet architecture that uses histogram equalization techniques.

## 3 Methodology

### 3.1 Proposed hybrid system

**Fig 3:**
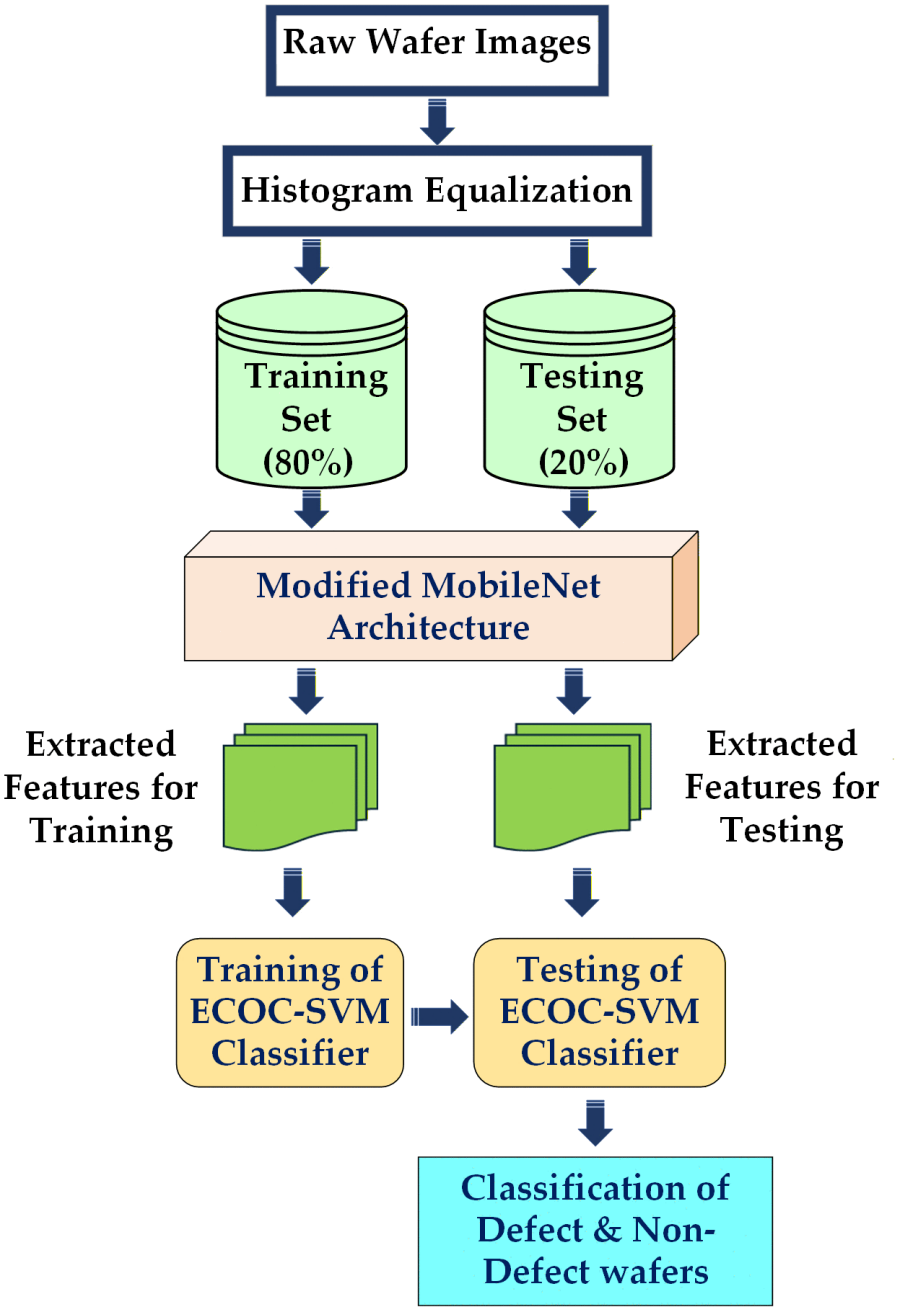
Methodology of the proposed hybrid system.

Existing wafer defect classification techniques [10,14] struggle to identify complex defective wafers and long-range correlations of defects in wafers, which negatively affect the performance of these systems. Also, developed techniques [9,10,12] have failed to detect small defects in wafers. To solve these limitations, this paper aims to design the modified MobileNet and ECOC-SVM-based hybrid system. The proposed method is depicted in Fig 3.

The process begins with the pre-processing step, in which the histogram equalization technique is applied to improve the visibility of minor defects in wafers. The MobileNet structure is modified by modifying the DSC block and integrating the multi-head attention mechanism into MobileNet. The modified architecture extracted features from training and testing wafer image sets. The features obtained from the training set are used to train the ECOC-SVM classifier. The features obtained from the testing set are fed into the trained ECOC-SVM classifier that classifies both defective and non-defective wafers. Finally, the proposed system’s performance is evaluated to identify wafer defects through performance metrics.

### 3.2 Dataset

**Fig 4:**
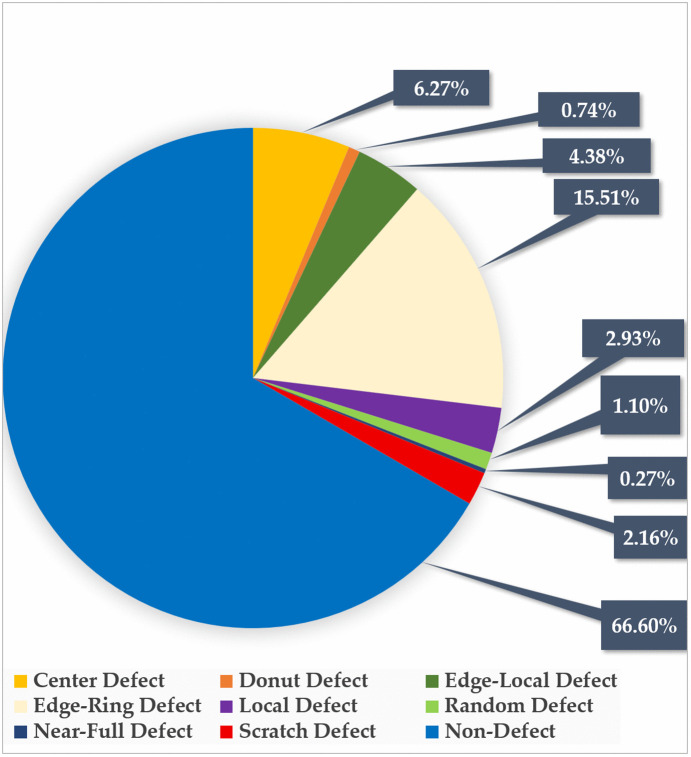
Distribution of Defect Classes in the Dataset.

This study investigates the classification of macro-scale wafer defect patterns by wafer map representations. The defect categories are ‘Center’, ‘Donut’, ‘Edge-Ring’, ‘Edge-Local’, ‘Scratch’, ‘Random’, ‘Near-Full’, and ‘Local’ defects. The widely accepted benchmark in the semiconductor manufacturing industry, the WM-811K [31] dataset, is used in this research. Many research studies use this data collection [9,10,12–14,20,25]. The pie chart of Fig 4 represents the overall distribution of defect classes. 55,143 real wafer map images are used in this study, among them 6.27% are ‘Center’ defect class, 0.74% are ‘Donut’ defect class, 4.38% are ‘Edge-Local’ defect class, 15.51% are ‘Edge-Ring’ defect class, 2.93% are ‘Local’ defect class, 1.10% ‘Random’ defect class, 0.27% are ‘Near-Full’ defect class, 2.16% are ‘Scratch’ defect class, and 66.60% are non- defect classes.

**Fig 5:**
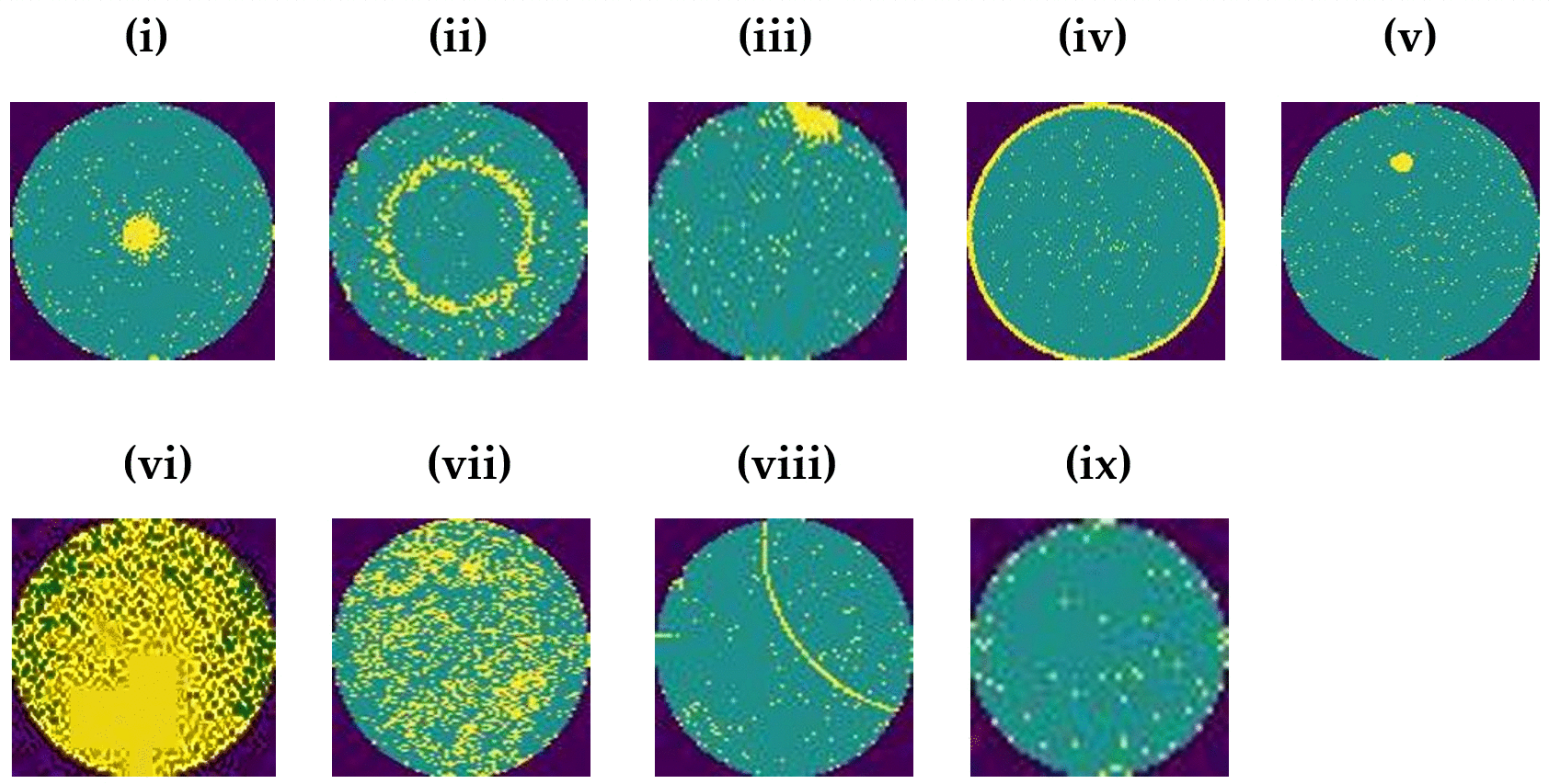
Defect and non-defect wafer map patterns before histogram equalization. Notes: (i) Center (ii) Donut (iii) Edge local (iv) Edge ring (v)Local (vi) Near full (vii) Random (viii) Scratch (ix) None.

Although there are imbalanced classes of defects and non-defect wafer images, the ECOC-based SVM classifier is used to mitigate this class imbalance problem. This classifier is discussed later in this paper. Fig 5 shows eight types of defect classes and one non-defect class. All of them are non-histogram equalized wafer map images.

### 3.3 Histogram equalization (HE) technique

Non-equalized defect wafer images exhibit inconsistent contrast because pixel intensity values are confined to a limited range; therefore, subtle features and tiny defects in wafer images are difficult to identify. For this reason, defective wafer detection systems encounter difficulties in identifying tiny defects in wafers.

The proposed method uses histogram equalization to address this problem. This technique distributes pixel intensity values to improve contrast in the wafer image. Pixel intensities in a defect wafer map usually indicate defect density; histogram equalization highlights the distinctions between defect and non-defect regions.

The pixel intensity value of the defect wafer map image is I(x,y), where x and y are the spatial coordinates on the wafer map. The total number of pixels in the wafer image is [32]:


$$T_{p}=M\times N$$ (1)

Where M and N are the dimensions of the wafer map. The intensity levels are redistributed using the cumulative distribution function (CDF). For a specific intensity level, the CDF is defined as [32]:


$$CDF\left(i\right)=\sum_{j=0}^{i}h\left(j\right)$$ (2)

Where h(j) is the number of pixels with intensity j. The normalized CDF is given by [32]:


$$CDF{\left(i\right)}_{norm}=\frac{CDF\left(i\right)}{T_{p}}$$ (3)

The total number of pixels is represented by ${\text{T}}_{\text{p}}$. The pixel values of the original image are then mapped to the new ones by transforming the pixel intensity values using the normalized CDF. With an intensity of I(x, y), the transformation function for that pixel is [32]:


$$I(x,y{)}_{new}=I_{min}+(CDF\left(i{)}_{norm}\left(I\left(x,y\right))\times (L-1\right)\right)$$ (4)

**Fig 6:**
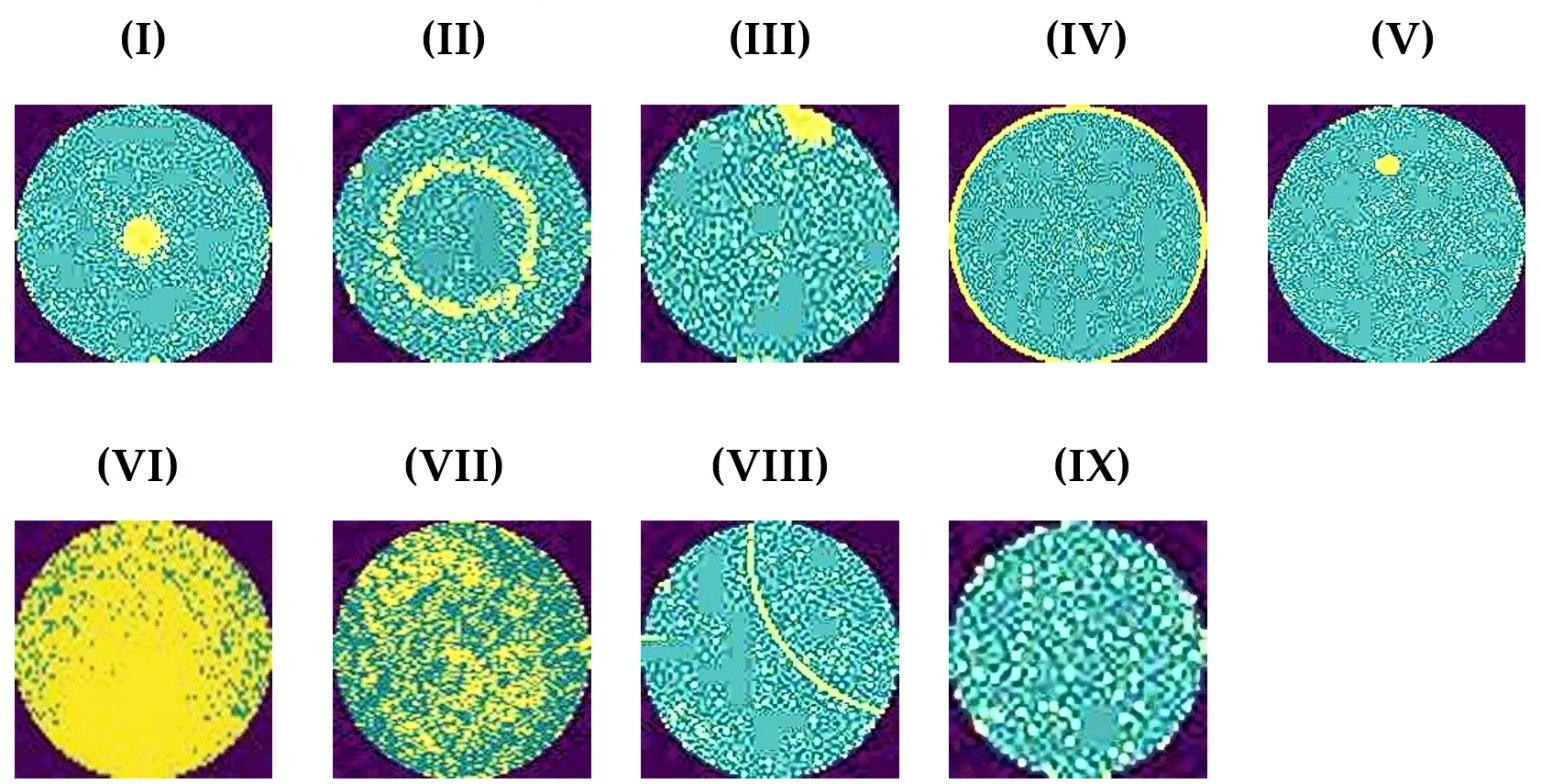
Defect and non-defect wafer map patterns after histogram equalization. Notes: (i) Center (ii) Donut (iii) Edge local (iv) Edge ring (v)Local (vi) Near full (vii) Random (viii) Scratch (ix) None.

Here, ${\text{I}}_{\text{min}}$is the minimum pixel intensity of the image, and L is the maximum possible intensity value. ${\text{I}(\text{x},\text{y})}_{\text{new}}$is the new intensity value after histogram equalization. The histogram equalized defective and non-defective wafer map images are illustrated in Fig 6. After applying histogram equalization techniques to the defective semiconductor wafers, the pixel intensity values of the defective region on the wafers are increased. As a result, the small defective features on the wafers are more visible than the non-equalized wafer images. This technique enables the model to accurately identify tiny defects, especially ‘Local’ defect patterns among the defective wafer categories, thus increasing the classification accuracy of the model.

**Fig 7:**
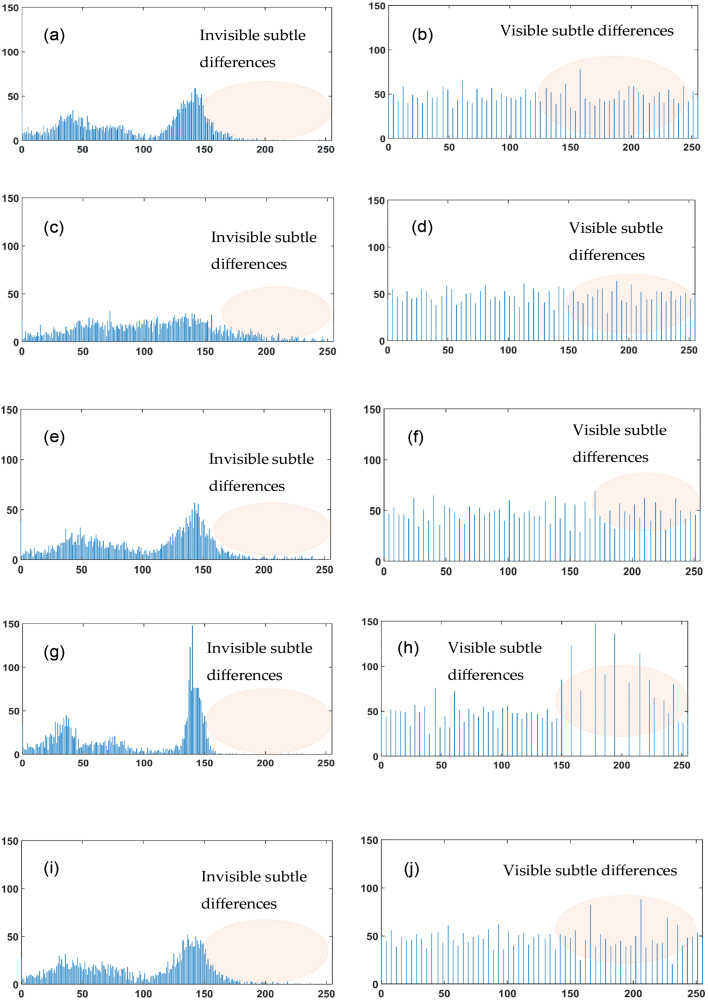
Histogram of equalized and un-equalized defective images. Notes: (a) histogram of unequalized Center defect image; (b) histogram of equalized Center defect image; (c) histogram of unequalized Edge-Local defect image; (d) histogram of equalized Edge-Local defect image; (e) histogram of unequalized Edge-Ring defect image; (f) histogram of equalized Edge-Ring defect image; (g) histogram of the unequalized Local defect image; (h) histogram of equalized Local defect image; (i) histogram of unequalized Scratch defect image; (j) histogram of equalized Scratch defect image.

Fig 7 illustrates the histogram of un-equalized and equalized wafer images, in which the x-axis denotes the range of intensity values & the y-axis denotes the number of pixels with a specific intensity value of the images.

The subtle differences in pixel intensities remain undetectable in the histogram of non-equalized defect images. The difference between defect areas and their surroundings becomes more apparent after the histogram equalization process. Fig 7(a), Fig 7(c), Fig 7(e), Fig 7(g), and Fig 7(i) represent the histograms of non-equalized images of ‘Center’, ‘Edge-Local’, ‘Edge-Ring’, ‘Local’, and ‘Scratch’ defective classes, respectively. The histograms indicate that most pixel intensities are clustered in the mid-range. This results in numerous complicated features within the defective region being diminished or turned invisible. Following the equalization of these defect images, the pixel intensities are redistributed more uniformly across the entire range shown in Fig 7(b), Fig 7(d), Fig 7(f), Fig 7(h), and Fig 7(j).

‘Local’ defect category exhibits small defective patterns. Fig 7(h) depicts the histogram of the equalized ‘Local’ defective patterns. The histogram of the equalized images shows pixel intensities distributed across a wider range. Also, these histograms show that spikes fluctuate between different heights, and pixel intensities vary from one region to another. These fluctuations reflect small variations in the intensities of the ‘Local’ defect image, which magnifies small defective regions in this defect class.

### 3.4 Proposed modified MobileNet architecture

The basic MobileNet model comprises the depth-wise separable convolution (DSC) block that uses the ReLU activation function. MobileNet architecture has lower parameter counts and computational costs. However, in detecting wafer defects, the MobileNet architecture confronts challenges in recognizing complex defective wafers. This paper presents a modified version of MobileNet, which modifies the DSC block by replacing the ReLU function with the Swish function to detect complex defective patterns. Additionally, the integration of the multi-head attention mechanism with the MobileNet architecture facilitates the identification of long-range spatial relationships of defects in wafers.

#### 3.4.1 MobileNet architecture.

**Fig 8:**
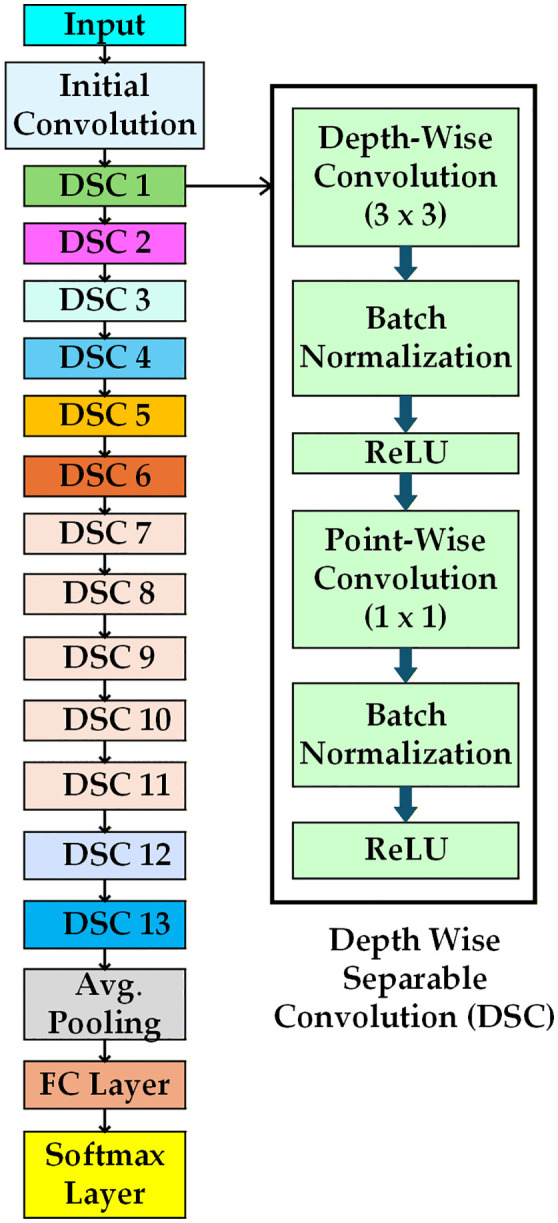
Structure of original MobileNet [28].

Fig 8 depicts the MobileNet architecture [28]. This structure comprises DSC blocks, global average pooling, the fully connected layer, and a classification layer using a softmax classifier. The fundamental components of the DSC block are proposed in [28].

However, MobileNet confronts problems in detecting complex defect patterns. MobileNet architecture utilizes the ReLU activation function in the DSC block. The ReLU function is expressed as [33]:


ReLU(x)=max(0,x) (5)

For positive values of x, ReLU outputs x (linear activation). For negative values of x, ReLU outputs 0 (non-linear, effectively deactivating the neuron). The derivative of the ReLU activation function is:


$$\text{for\textbackslash{}hspace\{0.33em}\;\}x<0,\text{\textbackslash{}hspace\{0.33em}\}\frac{d}{dx}ReLU\left(x\right)=0$$ (6)

$$\text{for\textbackslash{}hspace\{0.33em}\}x>0,\text{\textbackslash{}hspace\{0.33em}\}\frac{d}{dx}ReLU\left(x\right)=1$$ (7)

ReLU passes the gradient unchanged for positive inputs; on the other hand, for x< 0, the gradient of ReLU is zero, which means that during back propagation, the weights connected to these neurons do not get updated. This can lead to the “dying ReLU “ problem, where a significant portion of neurons stop learning, especially when defects are complex and involve subtle negative activations. For this reason, the MobileNet architecture cannot accurately identify complex defect wafers.

#### 3.4.2 Modified MobileNet architecture.

This paper proposes a modified MobileNet architecture to overcome the drawbacks of the original MobileNet architecture. The Swish function improves the modified MobileNet’s ability to identify intricate wafer defect patterns. The Swish function is defined as [34]:


$$Swish\left(x\right)=x.\sigma \left(x\right)=\frac{x}{1+e^{-x}}$$ (8)

**Fig 9:**
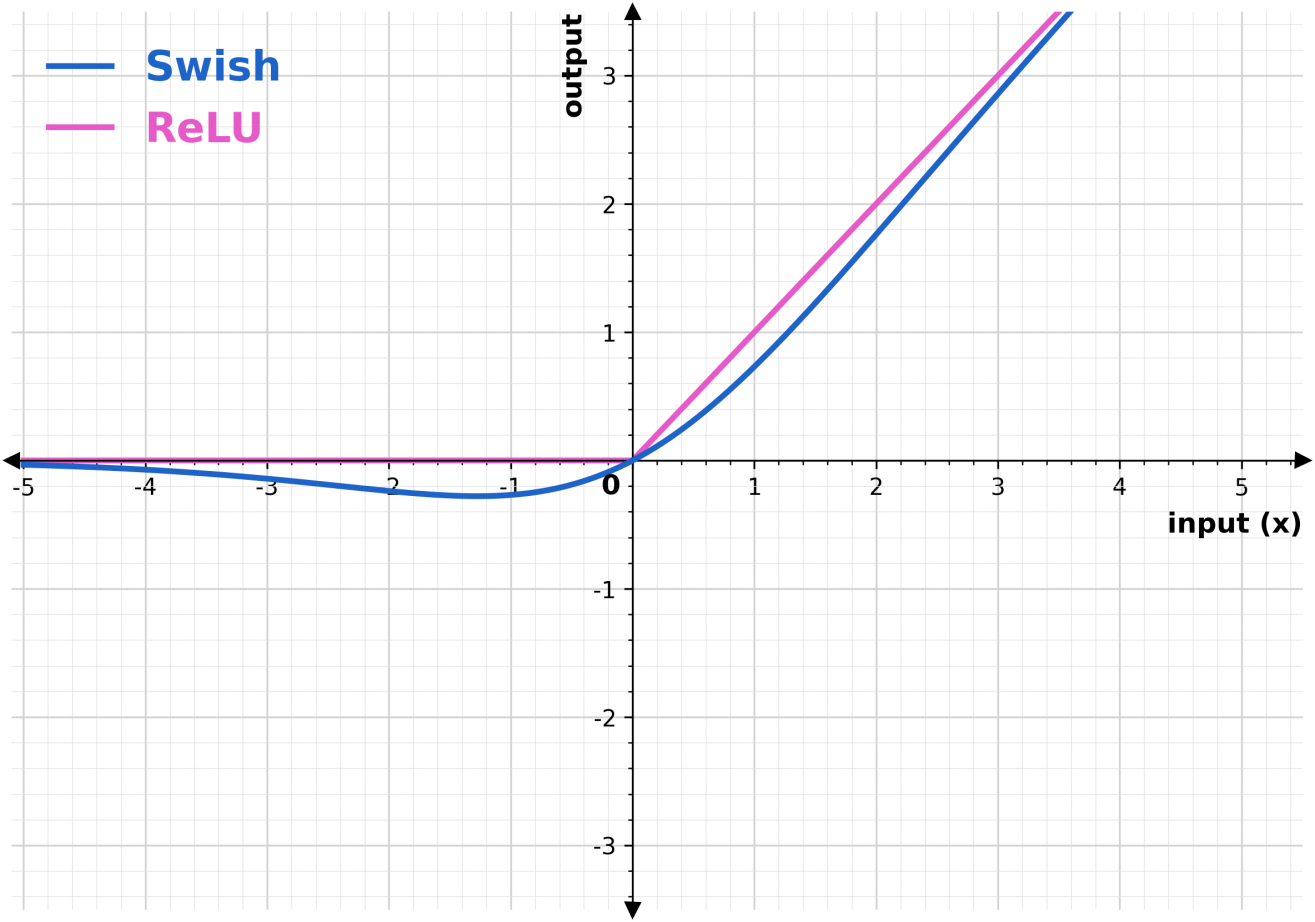
Graphical representation of ReLU and Swish activation functions.

Fig 9 depicts the difference between ReLU and Swish activation functions. The Swish function is a smooth, non-linear function that outputs values in the range (−∞, ∞). In Fig 9, for large positive values of input (x), the Swish is approximately linear, similar to ReLU. For large negative values of input (x), the Swish outputs a small negative value close to zero, but not exactly zero.

For values of input (x) around zero, the Swish function provides non-linear outputs, with a smooth transition from negative to positive input values. This smooth change enables the proposed MobileNet to respond to slight variations in defect image intensities, consequently facilitating the effective detection of complex defective patterns.

The derivative of the Swish function is:


$$\frac{d}{dx}Swish\left(x\right)=\sigma \left(x\right)+x.\sigma \left(x\right).\left(1-\sigma \left(x\right)\right)$$ (9)

**Fig 10:**
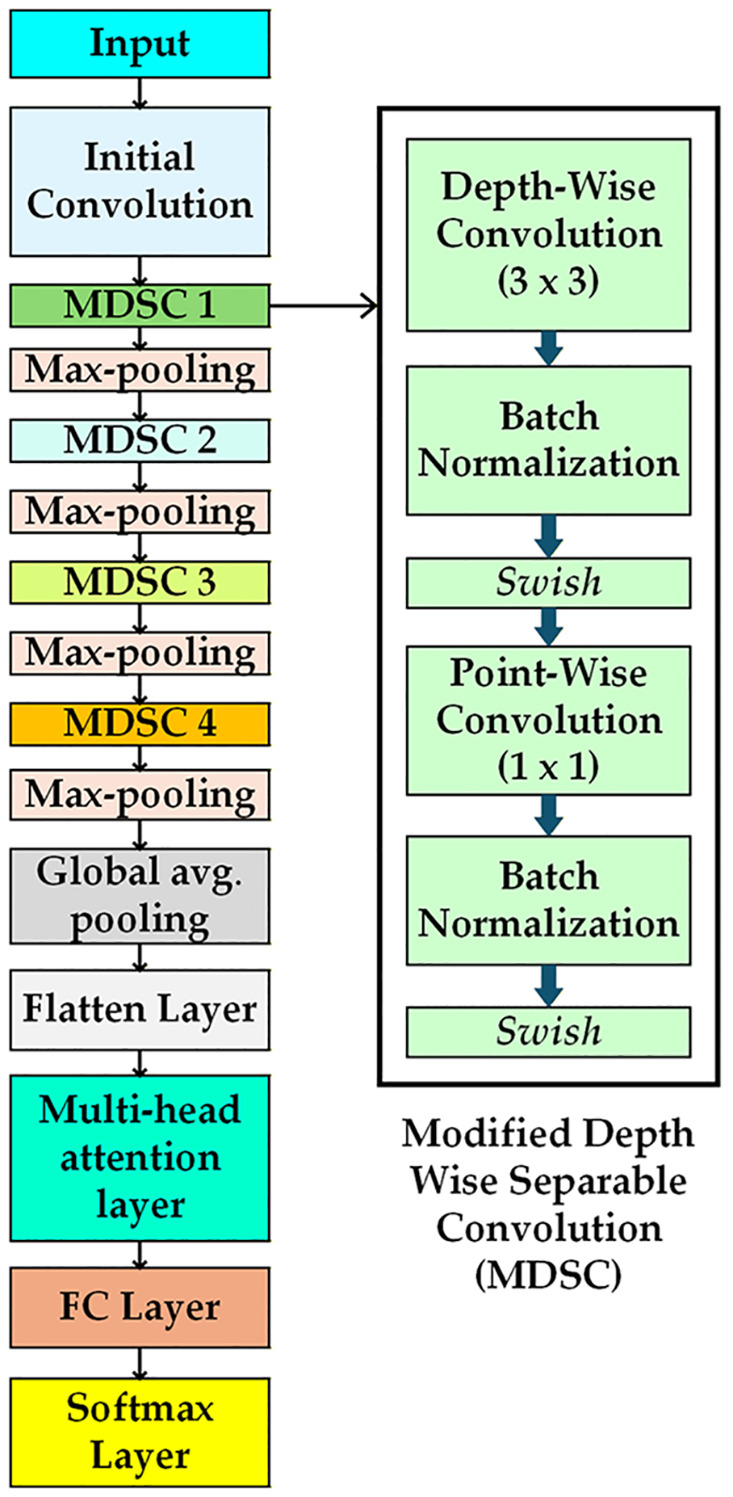
Structure of modifiedMobileNet [28].

Where σ(x) is the sigmoid function. This derivative is smooth and varies with input x, allowing for more minor updates to the weights during backpropagation. The term x. σ(x). (1-σ(x)) introduces non-linearities, which help the proposed model to extract features from complex patterns in defective wafers. These analyses indicate that for capturing complex defect wafers, the Swish activation function performs better than the ReLU activation function by adeptly collecting minor fluctuations in pixel intensities of histogram-equalized defect images, preserving a smooth gradient flow, and responding efficiently to input variations. Fig 10 illustrates the modified structure of MobileNet. This proposed architecture modifies the depth-wise separable convolution block, incorporating the Swish function instead of the ReLU function. The proposed architecture encompasses four MDSC blocks. Except for the Swish function, other elements of the MDSC block are identical to those of the DSC block of the original version of MobileNet. The max-pooling layers are strategically positioned following each MDSC to minimize spatial dimensions. Also, global average pooling is used in the modified MobileNet architecture to reduce the overall parameter count and computational complexity of the developed hybrid model.

However, this modified MobileNet architecture exhibits limitations in its ability to concentrate on distinct defect patterns within the wafers. Consequently, this architecture fails to capture the global context and the relationships among different parts of the defective wafer images, which reduces the overall effectiveness of the model in identifying wafer defects.

#### 3.4.3 Multi-head attention mechanism.

The limitation of the modified MobileNet model is solved by incorporating the multi-head attention mechanism into the modified architecture, which allows the hybrid model to effectively identify long-range spatial relations of defects, facilitating its ability to focus on relevant defect regions in the wafers. Also, this mechanism enables the modified architecture to measure the interactions between distant defective areas on the wafers. In multi-head attention mechanisms, the input feature maps of wafers are segmented into several smaller regions, referred to as heads. Each attention head functions autonomously, enabling the proposed model to identify various relationships and patterns in defective wafer feature maps. Each attention head comprises three distinct sets of parameter matrices: Query (Q), Key (K), and Value (V). Query, Key, and Value Vectors are given as [35]:


$$Q=XW^{Q}$$ (10)

$$K=XW^{K}$$ (11)

$$V=XW^{V}$$ (12)

**Fig 11:**
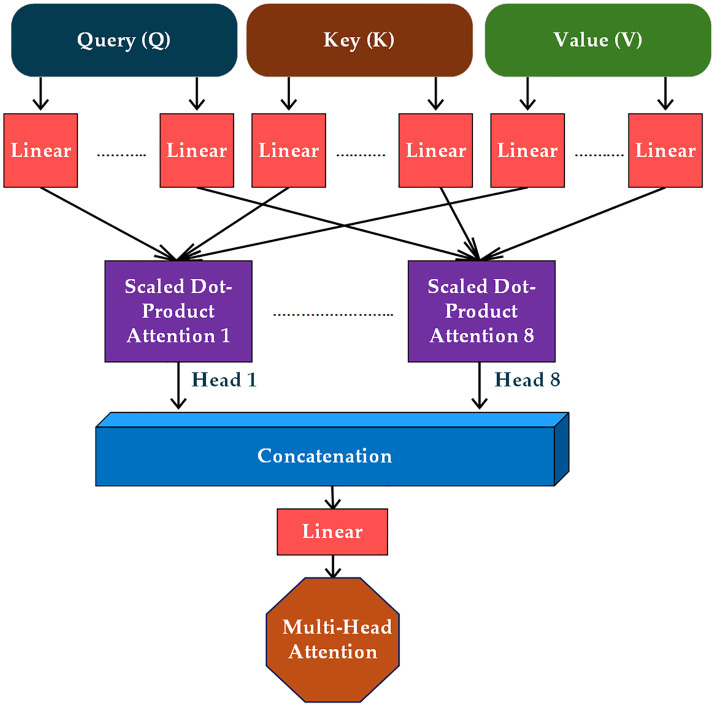
The multi-head attention mechanism process.

In this context, ${\text{W}}^{\text{Q}}$,${\text{W}}^{\text{K}}$and ${\text{W}}^{\text{V}}$represent the weight matrices that are learned, while X denotes the input. The Q denotes the feature representation of a specific region of the wafer map that the model emphasizes (current area),K indicates the feature representations of all regions (including the current and surrounding areas), and V encompasses the actual information to be aggregated according to the attention scores. The dimensionality of these matrices dictates the size of the vectors utilized for computing attention scores. Fig 11 depicts the process of the multi-head attention mechanism.

This illustration depicts the structure of a multi-head attention mechanism. We employ 8 heads in this mechanism. The input consists of three distinct components: Query (Q), Key (K), and Value (V). All these components undergo a linear transformation. The dimensions of the query and key are specified as ${\text{d}}_{\text{k}}$ while the dimension of the value is defined as ${\text{d}}_{\text{v}}$ [35]. Each component (Q, K, and V) is replicated 8 times, one for each attention component, namely Q,K, and V, and undergoes replication 8 times, corresponding to each attention head. After the linear transformations, the model systematically calculates the scaled dot-product attention for each head separately. This operation involves comparing the queries and keys, using the outcome to assign weights to the values, thereby enabling the model to focus on various areas of the defect wafers. The presence of 8 distinct attention heads arises from the advantage of employing multiple attention functions rather than relying on a singular attention mechanism with ${\text{d}}_{\text{model }}$ dimensional keys, values, and queries. Each head independently calculates its attention weights, allowing the model to gather contextual information from diverse representations of defect wafers across multiple positions. The outputs from all 8 attention heads are concatenated, effectively integrating the information derived from each head. Subsequently, an additional linear layer processes the combined outputs to generate the ultimate multi-head attention output. The multi-head attention mechanism calculates the attention score for each region in the wafer map corresponding to every defect pattern in the following equation [35]:


$$Attention\left(Q,K,V\right)=Softmax\left(\frac{QK^{T}}{\sqrt{d_{K}}}\right)V$$ (13)

The softmax operation serves to normalize the attention weights in this context. This scaled dot product attention emphasizes the relevant parts of the wafer map while diminishing the impact of irrelevant regions. Once the attention is calculated for each head, the outputs from all heads are combined through concatenation as follows [35]:


$$Output=Concat\left(head_{\text{1}},head_{\text{2}},...,head_{\text{8}}\right)W^{O}$$ (14)

Where,


$$head_{i}=Attention\left(Q\overset{Q}{\underset{i}{W}},K\overset{K}{\underset{i}{W}},V\overset{V}{\underset{i}{W}}\right)$$ (15)

Here, i=1.....8

And,


$$\overset{Q}{\underset{i}{W}}\in R^{d_{Model}\times d_{K}}$$ (16)

$$\overset{K}{\underset{i}{W}}\in R^{d_{Model}\times d_{K}}$$ (17)

$$\overset{V}{\underset{i}{W}}\in R^{d_{Model}\times d_{V}}$$ (18)

$$W^{O}\in R^{d_{Model}\times nd_{V}}$$ (19)

Where ${\text{W}}^{\text{O}}$ is the final transformation weight matrix. In this proposed model, the ${\text{d}}_{\text{Model}}$ is 512, and the multi-head attention mechanism has 8 heads. Now, ${\text{d}}_{\text{k}}$ and ${\text{d}}_{\text{v}}$are computed as:


$$d_{k}=d_{v}=\frac{d_{Model}}{n}=\frac{512}{\text{8}}=64$$

The overall computational cost is similar to single-head attention with full dimensionality because each head has a smaller dimension [35].

**Fig 12:**
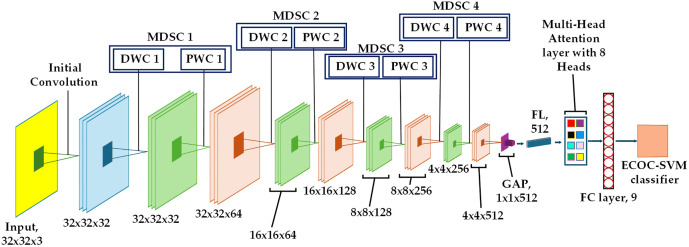
The detailed architecture of the proposed hybrid model.

The proposed hybrid architecture is illustrated in Fig 12. The multi-head attention in the proposed MobileNet allows the model to find the interactions among distant defect regions on the wafer. The proposed method replaces the modified MobileNet architecture classification layer with the ECOC-SVM classifier to build a hybrid system. This architecture leverages four modified depth-wise separable convolution (MDSC) blocks. MDSC blocks comprise a depth-wise convolution (DWC) and a pointwise convolution (PWC). Depth-wise convolution requires a separate filter for each input channel, reducing computational demands. Point-wise convolution uses 1x1 convolutions to combine channel-wise features, increasing the model’s depth. All DWC and PWC include batch normalization and swish activation functions.

Max-pooling (MP) is applied with a 2x2 size after each MDSC block to reduce spatial dimensions and improve the receptive field. The input images, sized 32x32 pixels with 3 color channels, are processed by the initial convolution layer. This layer identifies key image features and generates 32x32x32 feature maps. In the MDSC1 block, spatial filtering and channel combination are divided by a DWC featuring 32 filters of size 3x3, reducing computational cost. DWC 1 outputs are directed to PWC 1. The 1x1 convolution integrates outputs from the DWC by combining channel information with 64 filters, resulting in feature maps of size 32x32x64. This feature is reduced to 16x16x64 via max pooling. DWC 2 of the MDSC 2 block employs 64 filters to enhance each channel’s spatial feature, while PWC 2 combines the 64-channel output, resulting in 128 feature maps of 16x16 dimensions using 128 filters. The max pooling operation is performed after the MDSC 2 block, down sampling the feature maps by a factor of 2 to dimensions of 8x8x128. MDSC 3 produces 256 feature maps of size 4 × 4, using 128 filters in DWC and 256 filters in PWC. MDSC4 blocks generate 512 feature maps, each measuring 2x2, following the max pooling operation. The MDSC4 features DWC with 256 filters and PWC with 512 filters. This architecture uses global average pooling (GAP) to reduce the spatial dimension of 2x2x512 to a single vector of size 1x1x512 by averaging features across each channel. The flatten layer (FL) converts the GAP layer’s output into a one-dimensional vector of 512 features. It greatly reduces the total parameter count. The FL output is passed to the multi-head attention layer. This layer utilizes attention mechanisms with 8 heads and an output dimension of 64. This architecture employs two fully connected (FC) layers. The output of the multi-head attention layer connects to a fully connected layer with 9 neurons, representing the total classes of defect and non-defect wafer maps. Features from both the training and testing sets are extracted from this fully connected layer.

#### 3.4.3 ECOC technique-based SVM classifier.

SVM acts as a supervised ML algorithm specifically designed for binary classification tasks. The method involves directly identifying a hyperplane to separate two classes. The primary goal of SVM is to optimize the distance between each class’s closest data points (support vectors) and the hyperplane. However, the multiclass classification problems cannot be handled using SVM alone. To solve this issue, the proposed method utilizes the ECOC-based SVM classifier. This classifier classifies eight defect classes and one non-defect class, and solves the class imbalance problem.

The ECOC method integrates binary SVM classifiers to address multiclass issues [36]. The ECOC technique transforms a multi-class classification problem into several binary classification problems. This decomposition addresses class imbalance by directing each binary classifier’s attention towards specific class pairs, redistributing focus and mitigating the effects of imbalance within the framework of each binary problem. The ECOC–SVM technique comprises the ECOC encoding and the ECOC decoding stages. A coding strategy is necessary to train SVM binary learners during the encoding stage. In this study, the one vs one coding design [37] is used for the categorization of defect classes. Instead of learning the entire multi-class issue, each binary classifier learns to determine whether a wafer image belongs to a subset of defect classes during training. This code’s error-correcting feature adds redundancy, improving the classifier’s ability to overcome the class imbalance problem.

**Fig 13:**
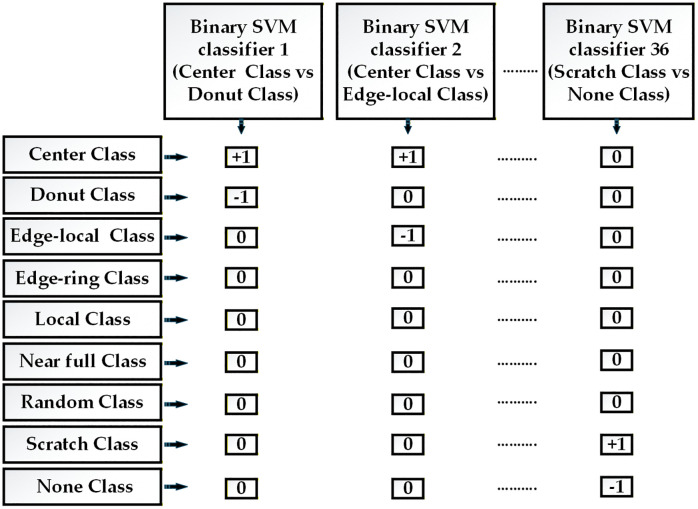
The block diagram of the ECOC coding matrix.

The final classification of defect and non-defect classes is conducted during the decoding stage by combining the outputs of all binary SVM classifiers. The total number of binary SVM classifiers for a one-versus-one structure with nine classes is calculated as follows:


$$\text{Total\textbackslash{}hspace\{0.33em}no.\;of\;binary\;classifiers\}=\frac{n(n-1)}{\text{2}}=\frac{9(9-1)}{\text{2}}=36$$

where n (n = 9) is the number of classes. To differentiate between the two classes, a total of 36 binary SVM classifiers are trained. Nine rows, one for each class, and thirty-six columns, one for each binary classifier, make up the ECOC code matrix. Fig 13 shows the block diagram of the ECOC code matrix; with +1 denoting that the class is the positive class for that binary classifier, −1 denoting that the class is the negative class for that binary classifier, and 0 denoting that the class is excluded from this binary classification task, each entry in the code matrix is set to +1, −1, or 0. A binary SVM classifier is trained for every column in the code matrix to distinguish between the two classes with non-zero values. The training process for each SVM is as follows: For a given classifier, given a feature vector x and binary labels y ε {+1,−1}.

The SVM maximizes the margin between the two classes to choose the best hyperplane that separates them. Each SVM classifier solves the following optimization problem to determine the best separation hyperplane between the two classes in the following manner [1]:


$$\underset{w,b,\xi }{min}0.5{\|w\|}^{\text{2}}+C\sum_{i=0}^{N}\xi_{i}$$ (20)

Subject to:


$$y_{i}\left(w.x_{i}+b\right)\geq 1-\xi_{i}$$ (21)

Where w is the weight vector, b is the bias,$\xi_{i}$ is slack variables, C is the regularization parameter, and ${\text{y}}_{\text{i }}$ is the label derived for each binary classifier. The SVM assigns a label of +1 or −1 to each test sample, determined by its position relative to the hyperplane. To classify a new defect class x, each of the 36 binary SVM classifiers outputs either +1 or −1, based on the position of x relative to the hyperplane. This output is a 36-bit code for the specific defect class. The output code is compared with each code word in the coding matrix, and the defect class linked to the code word with the closest distance to the output code is identified as the predicted defect class. The decoding stage uses the Hamming distance to find the nearest distance.

## 4 Result analysis

The experiment is performed using a laptop equipped with the 11th Generation Intel Core i5-1135G7 Processor. The hardware resource involves a single CPU. The MATLAB R2023a version is selected for this experiment. The modified lightweight MobileNet architecture utilized global average pooling, which enabled adequate CPU-based experiments on the dataset WM-811k [31]. Table 1 summarizes the implementation details of the proposed system.

**Table 1:** Experimental Settings.

Input image size	32 × 32
**Optimizer**	SGDM
**Learning rate**	0.001
**Batch Size**	128
**No. of Epochs**	10

### 4.1 Performance evaluation

The overall performance of the proposed system is demonstrated in this section using performance matrices and various optimizers. All experiments are performed according to the details in Table 1 on the wafer map dataset [31] with the imbalanced class distribution.

#### 4.1.1 Performance analysis of proposed system.

Table 2 presents the performance study of the proposed model, using five performance indicators: accuracy, precision, recall, AUC, and F1 score.

**Table 2:** Performance analysis of proposed hybrid model configurations on the imbalanced WM-811k dataset (image size:32 × 32).

Model	Measure	Center	Donut	Edge –Local	Edge -Ring	Local	Near Full	None	Random	Scratch	Avg.
Original MobileNet with ReLU (Base model) + HE + Softmax	Precision	0.931	0.936	0.891	0.988	0.700	0.985	0.980	0.958	0.432	0.867
	F1-Score	0.952	0.682	0.840	0.989	0.678	0.985	0.983	0.844	0.466	0.824
	Recall	0.975	0.536	0.795	0.990	0.657	0.985	0.987	0.754	0.506	0.798
	AUC	0.997	0.875	0.993	0.999	0.971	0.985	0.995	0.949	0.945	0.968
Modified MobileNet Model with Swish + HE+ ECOC-SVM	Precision	0.958	0.907	0.943	0.880	0.882	0.923	0.965	0.912	0.630	0.889
	F1-Score	0.955	0.915	0.929	0.904	0.848	0.914	0.962	0.922	0.605	0.884
	Recall	0.952	0.9231	0.916	0.9297	0.835	0.905	0.958	0.932	0.582	0.881
	AUC	0.943	0.9558	0.9351	0.9647	0.958	0.958	0.948	0.9439	0.9033	0.945
Modified MobileNet Model with Swish + HE+Multi-HeadAttention+Softmax	Precision	0.965	0.832	0.874	0.985	0.775	0.860	0.979	0.823	0.597	0.854
	F1-Score	0.969	0.7846	0.840	0.986	0.713	0.809	0.980	0.886	0.429	0.822
	Recall	0.974	0.741	0.809	0.988	0.660	0.733	0.991	0.959	0.334	0.799
	AUC	0.962	0.844	0.956	0.934	0.943	0.794	0.986	0.979	0.884	0.920
Modified MobileNet Model with ReLU + HE +Multi-head Attention + ECOC-SVM	0.976	0.904	0.899	0.914	0.899	0.978	0.971	0.881	0.692	0.902	0.976
	0.969	0.906	0.897	0.910	0.837	0.925	0.974	0.894	0.638	0.883	0.969
	0.960	0.9082	0.8952	0.9072	0.7834	0.8779	0.9772	0.9083	0.5928	0.868	0.960
	0.943	0.908	0.949	0.936	0.926	0.908	0.969	0.958	0.861	0.929	0.943
Modified MobileNetModel with Swish + Multi-Head Attention+ ECOC-SVM (Proposed Hybrid Model)	Precision	0.985	0.951	0.966	0.997	0.915	**1.000**	0.991	0.944	0.855	**0.956**
	F1-Score	0.990	0.951	0.960	**0.996**	0.894	0.947	0.994	0.955	0.807	**0.944**
	Recall	0.995	0.951	0.954	**0.995**	0.873	0.990	0.907	0.967	0.765	**0.933**
	AUC	0.999	0.999	0.998	0.999	0.994	**1.000**	0.996	0.999	0.987	**0.997**

The AUC values demonstrate the model’s capacity to recognize intricate patterns in semiconductor wafer maps. The optimal results in this table are defined in bold font. This table shows the F1-score, precision, recall, and AUC metrics for eight defect categories and one non-defect category. The proposed hybrid system attains the greatest average F1-score of 0.933 among all model configurations in this table. The proposed hybrid approach outperforms the baseline MobileNet model’s overall precision and AUC values, increasing by 8.9% and 2.9%, respectively, which demonstrates the hybrid method’s efficacy in detecting complex defective patterns and tiny defects in wafers. The overall recall value significantly increases from 0.798 (using original MobileNet) to 0.944 (using proposed model),demonstrating that the suggested approach effectively mitigates the class imbalance issues of the dataset [31] by concentrating on the minority defective classes. The Swish-based proposed system surpasses the ReLU-based MobileNet model in recognizing ‘Donut’, ‘Edge-ring’, and ‘Scratch’ types of intricate defect patterns, boosting the F1-score and AUC values for these defective wafer categories. The proposed model achieved superior efficiency in recognizing long spatial defect linkages in wafers, evidenced by a substantial gain in precision by 42.3%for the ‘Scratch’ defect class and a boost in AUC value by 12.4% for the ‘Donut’ defect class compared to the original MobileNet model. Despite the modest gain in recall metric for the ‘Near-Full’ defect class in the minority group of datasets [31], other classes, such as ‘Donut’, ‘Random’, and ‘Scratch’, exhibit significant improvements in recall values of 41.5%, 21.3%, and 25.9%, respectively, by leveraging the proposed model with ECOC-SVM classifier in comparison to the base model with softmax classifier.

#### 4.1.2 Performance analysis with different optimizers.

**Fig 14:**
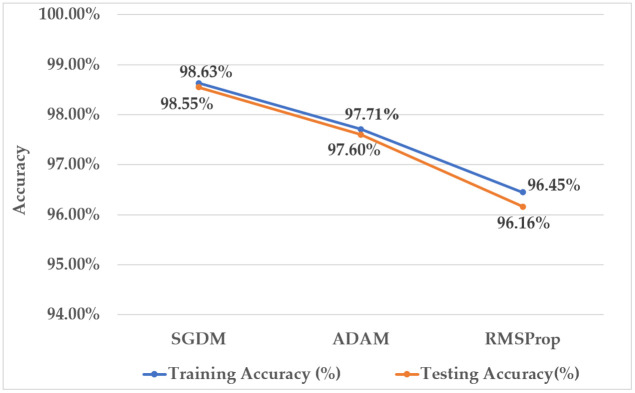
Analysis of the proposed model using several optimizers.

Three optimizers are employed for performance observation of the proposed model: ‘SGDM’, ‘ADAM’, and ‘RMSProp’ optimizers. The Stochastic Gradient Descent with Momentum (SGDM) optimizer minimizes computational redundancy [38]. The momentum parameter of this optimizer facilitates the acceleration of the optimization process. The Root Mean Square Propagation (RMSProp) optimizer employs the concept of a moving average of the squared gradients across consecutive mini-batches for each weight [39]. The Adaptive Moment Estimation (ADAM) optimizer integrates the properties of AdaGrad and RMSProp and has lower memory [40]. Fig 14 presents a line graph comparing the performance of the proposed hybrid system using three distinct optimizers.

The suggested system with the SGDM optimizer achieved a testing accuracy of 98.55%, exceeding the accuracy with the RMSProp optimizer by 2.93%. Furthermore, in wafer defect identification, the model’s accuracy improved with the utilization of the SGDM optimizer compared to the ADAM optimizer.

### 4.2 Ablation study

This section analyses the efficacy of each modification element utilizing the given experimental setup with conditions of unbalanced class distribution in the dataset [31]. The ablation analysis is performed by removing each architectural component from the proposed hybrid design.

#### 4.2.1 Impact of Swish function.

**Fig 15:**
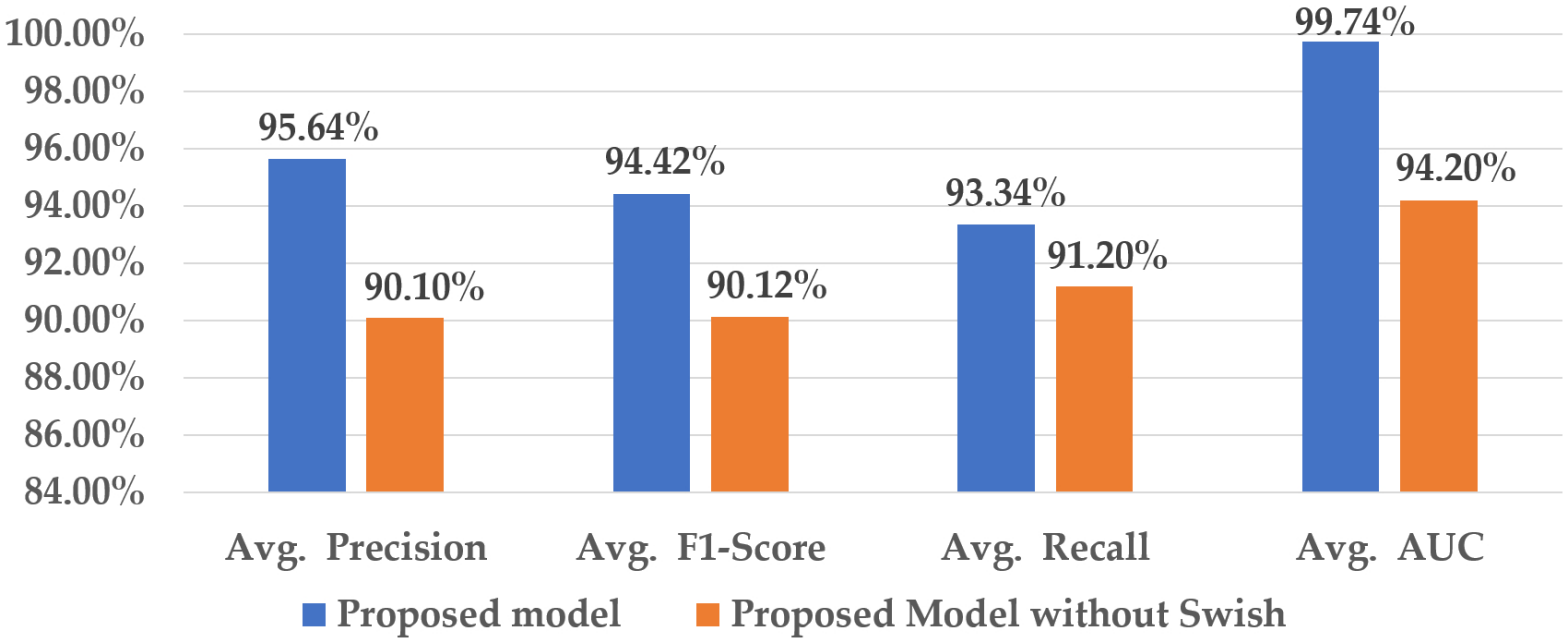
Contribution of the Swish activation function to the proposed model.

Fig 15 demonstrates the efficacy of the Swish activation function within the proposed system.

This analysis employs the ReLU function in place of the swish function within the hybrid system. Although the elimination of the swish function has less impact on the recall value, this removal led to a decrease in average precision to 90.10%, and the average F1-score of the model dropped by more than 5%. A notable decrease is observed in the overall AUC, from 99.74% to 94.20%. These outcomes indicate that the lack of the swish function renders the hybrid model less effective in identifying complex fault patterns. The non-linear characteristics of the swish function make it more suitable for the proposed model to detect intricate defective patterns. This activation function allows the hybrid model to identify subtle intensity variations in wafer defect images.

#### 4.2.2 Impact of multi-head attention.

**Fig 16:**
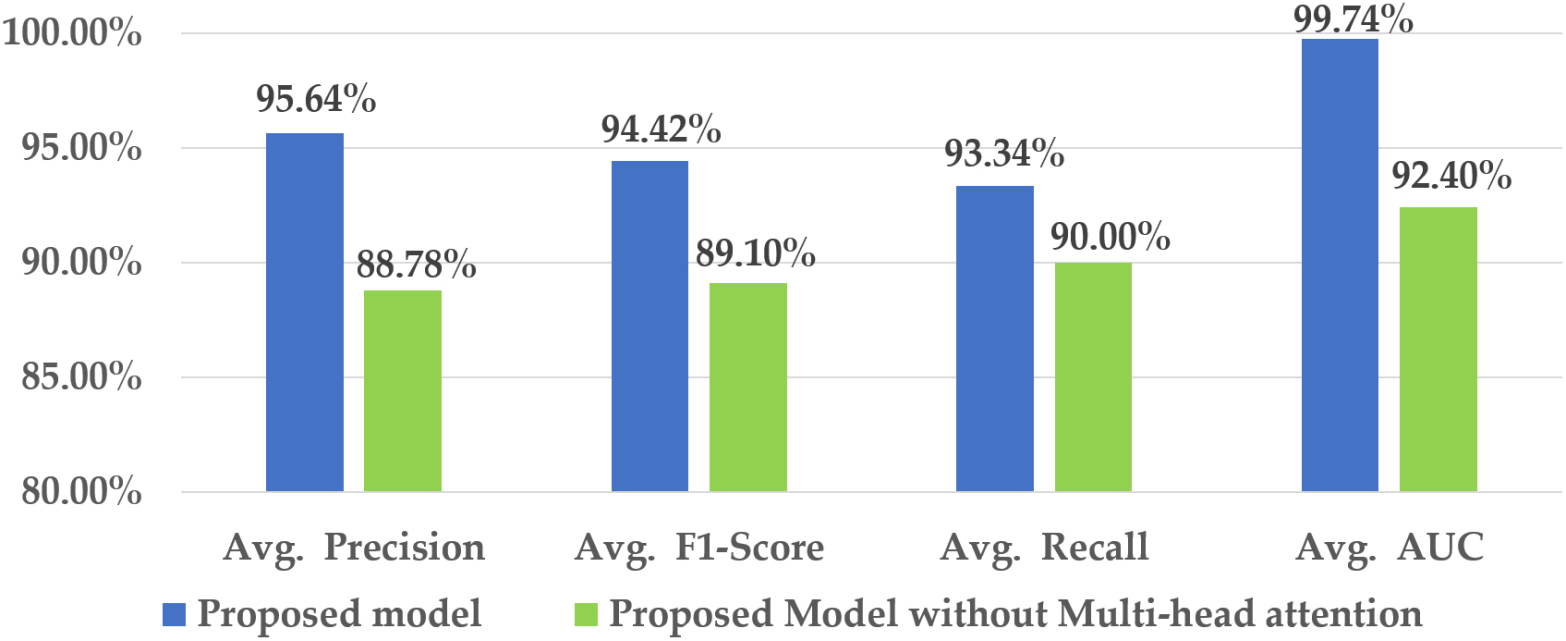
Contribution of the multi-head attention to the proposed model.

The statistical evaluation of the multi-head attention mechanism’s role in the proposed hybrid approach is illustrated in Fig 16.

The elimination of multi-head attention from the hybrid model results in a reduction in the average precision value from 95.64% to 88.78%. Additionally, the average AUC value notably declines by 7.34%, and the F1-score drops to 89.10%. These outcomes indicate the hybrid model’s inefficiency in recognizing long spatial relationships in wafer defects without the multi-head attention mechanism. The multi-head attention strategies capture spatially distant defective regions by using the attention heads, which enables the model to identify long-range defect patterns. The decrease in recall value is lower than that of other performance indicators.

#### 4.2.3 Impact of the ECOC-SVM classifier.

**Fig 17:**
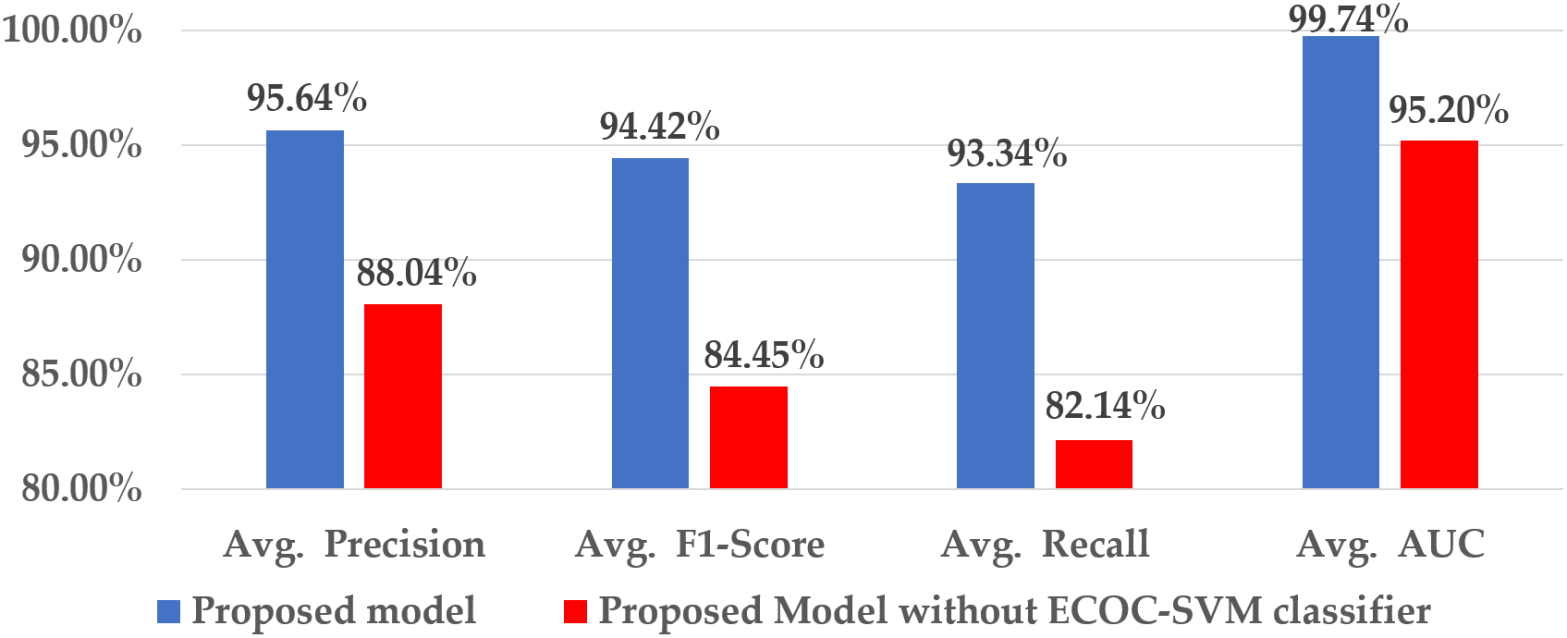
Contribution of the ECOC-SVM classifier to the proposed model.

Fig 17 illustrates the effectiveness of the ECOC-SVM classifier in addressing the class-imbalance problem.

The softmax classifier is employed in the hybrid model instead of the ECOC-SVM classifier for statistical analysis. Excluding the ECOC-SVM classifier, the recall value is drastically reduced by 11.2%, indicating that the model is incapable of handling class imbalance issues. The ECOC technique enables the hybrid model to concentrate on minority defect classes rather than exhibiting bias towards majority classes. This modification results in a 7.6% decrease in the average precision value, and the overall F1-score drops from 94.42% to 84.45%. The removal of the ECOC-SVM classifier from the hybrid model has a lesser effect on the AUC value compared to other performance metrics.

### 4.3 Effects of histogram equalization on the proposed hybrid model

**Fig 18:**
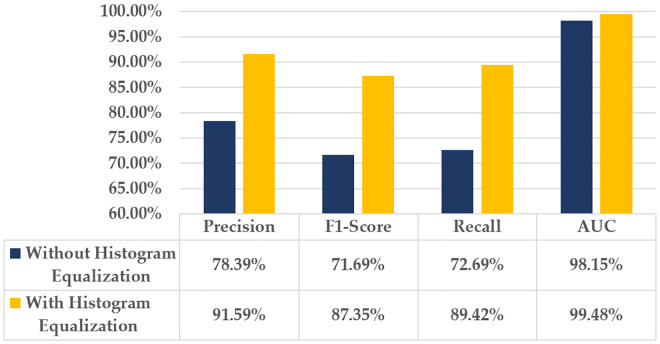
Impact of histogram equalization on ‘Local’ defect class.

This section discusses the crucial role of histogram equalization in identifying tiny defects in defective wafers. Among the eight types of defects, ‘Local’ is characterized as a small defect pattern. Fig 18 illustrates the impact of using histogram equalization on the proposed model’s ability to recognize the ‘Local’ defect class.

Precision value increased by 13.2% with histogram equalization, indicating improved accuracy in true positive predictions. The F1-Score substantially increases from 71.69% to 87.35%, indicating improved precision-recall balance. Recall value raised to 89.42%, demonstrating a better rate of accurately identified ‘Local’ defect class. This processing technique slightly increases the AUC value.

**Fig 19:**
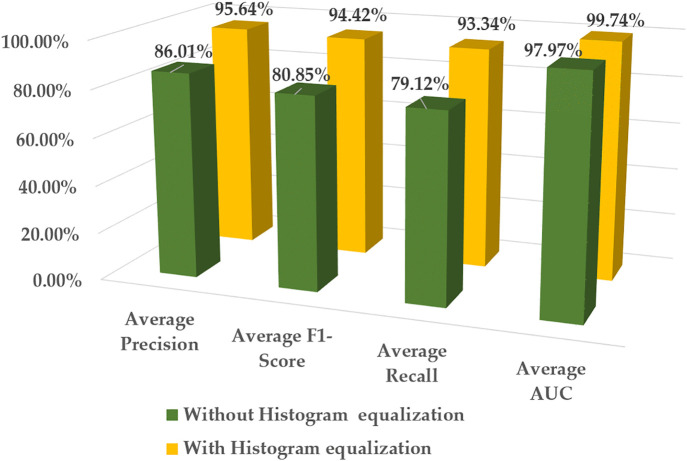
Overall performance evaluation of the proposed model based on the histogram equalization process.

Fig 19 illustrates a Column chart that examines the impact of applying the histogram equalization technique on the overall performance of the proposed hybrid model using the defective wafer dataset WM-811k [31].The results indicate that histogram equalization markedly improves the performance of the proposed model, with a 9.63% increase in average precision.

The histogram equalization technique boosts the average F1-Score of the hybrid model from 80.85% to 94.42%, demonstrating its efficacy in enabling the proposed model to detect subtle variations in the intensities of defective wafer images. Furthermore, the AUC value has a slight increase; the hybrid model’s average recall value rises to 93.34% with the implementation of histogram equalization.

### 4.4 The confusion matrix of the proposed hybrid system

**Fig 20:**
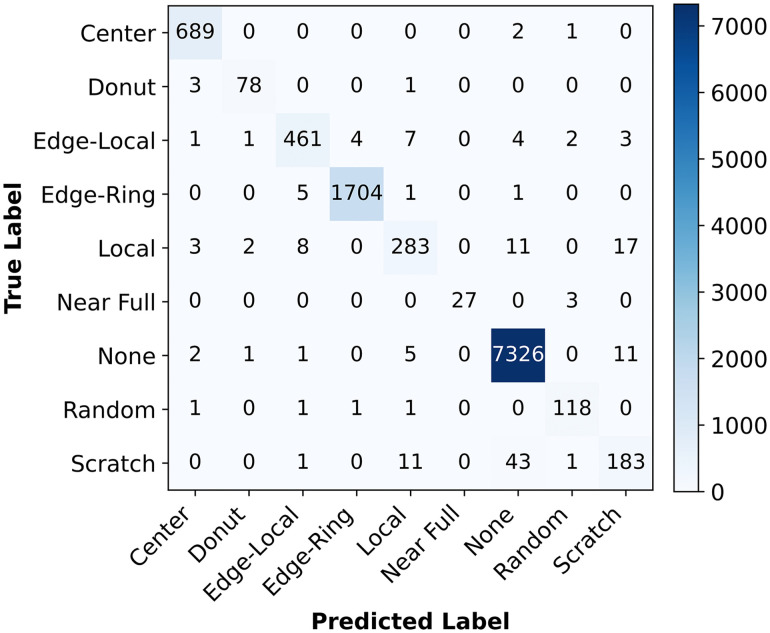
Confusion matrix of the proposed model.

This paper analyzes the effectiveness of the proposed hybrid approach using a confusion matrix, as depicted in Fig 20, which indicates the number of correctly classified defect wafer classes and the misclassification rate by the proposed model.

The diagonal entries of the confusion matrix represent the number of correctly classified images for each defect class, while off-diagonal entries indicate misclassifications between defect classes. The 689 of the ‘Center’ defect images are correctly classified, as shown in the starting row and first column of the confusion chart. Also, the value 1 in the first row and the 8th column in this chart signifies one image from the defect class center misclassified as the defect class ‘Random’ (false positives). The suggested model shows strong performance in identifying edge-ring defects, indicated by the few images classified as ‘Edge-Local’ and ‘Local’ defect classes. These outcomes indicate a markedly higher rate of correctly classified defect classes than the misclassification rate. However, the developed model in this paper has lower classification accuracy for the ‘Scratch’ defect class, due to higher misclassification rates compared to other classes, as indicated by the confusion matrix.

### 4.5 Comparative study of the proposed model using different classifiers

This study utilized several classifiers to identify defective wafers, as shown in Table 3 under conditions of the defect class-imbalanced dataset [31].

**Table 3:** Performance analysis of the proposed hybrid model based on different classifiers.

Classifiers	Overall Precision	Overall Recall	Overall F1-Score	Testing Accuracy
Softmax	88.04%	82.14%	84.45%	95.62%
KNN	76.32%	74.52%	75.33%	93.23%
ECOC-SVM	95.64%	93.34%	94.42%	98.55%

The hybrid model with the ECOC-SVM classifier achieves the highest precision value (95.64%), surpassing the softmax classifier by 7.6% and the KNN classifier by 19.32%. These outcomes indicate that the proposed classifier effectively reduces false positives, which is vital for accurate classification. The highest overall recall value is attained by the classifier used in this study, compared with other classifiers. The ECOC-SVM classifier model outperforms the softmax classifier by 9.97% and the KNN classifiers by 19.09% improvement in the overall F1 score. Also, the proposed classifier model attained higher accuracy in wafer defect pattern classification than other classifiers listed in this table, utilizing the experimental setup in Table 1.

### 4.6 Comparative analysis of the proposed model with state-of-art models

**Fig 21:**
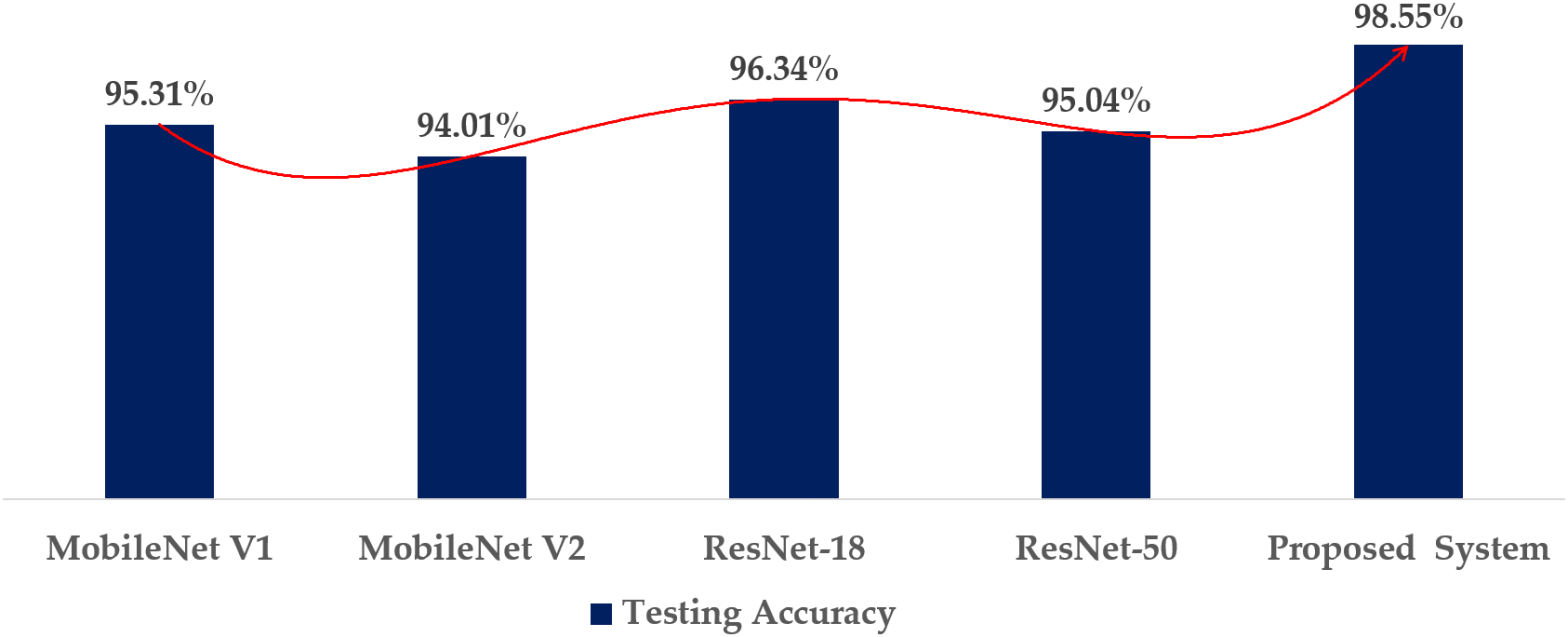
Comparative analysis based on testing accuracy.

All models in this section, including the proposed model, are evaluated under similar experimental settings described in Table 1 and the imbalanced dataset WM-811k [31].The bar chart in Fig 21 illustrates the testing accuracy of various deep learning models, including two variations of MobileNet, two variants of ResNet, and a proposed model.

The testing accuracy of the proposed model exceeds MobileNet V1andMobileNet V2 by 3.24% and 4.54%, respectively, which proves that the suggested modified MobileNet performs better than the two MobileNet versions. Also, the proposed model surpassed ResNet-18 and ResNet-50 in terms of accuracy.

**Fig 22:**
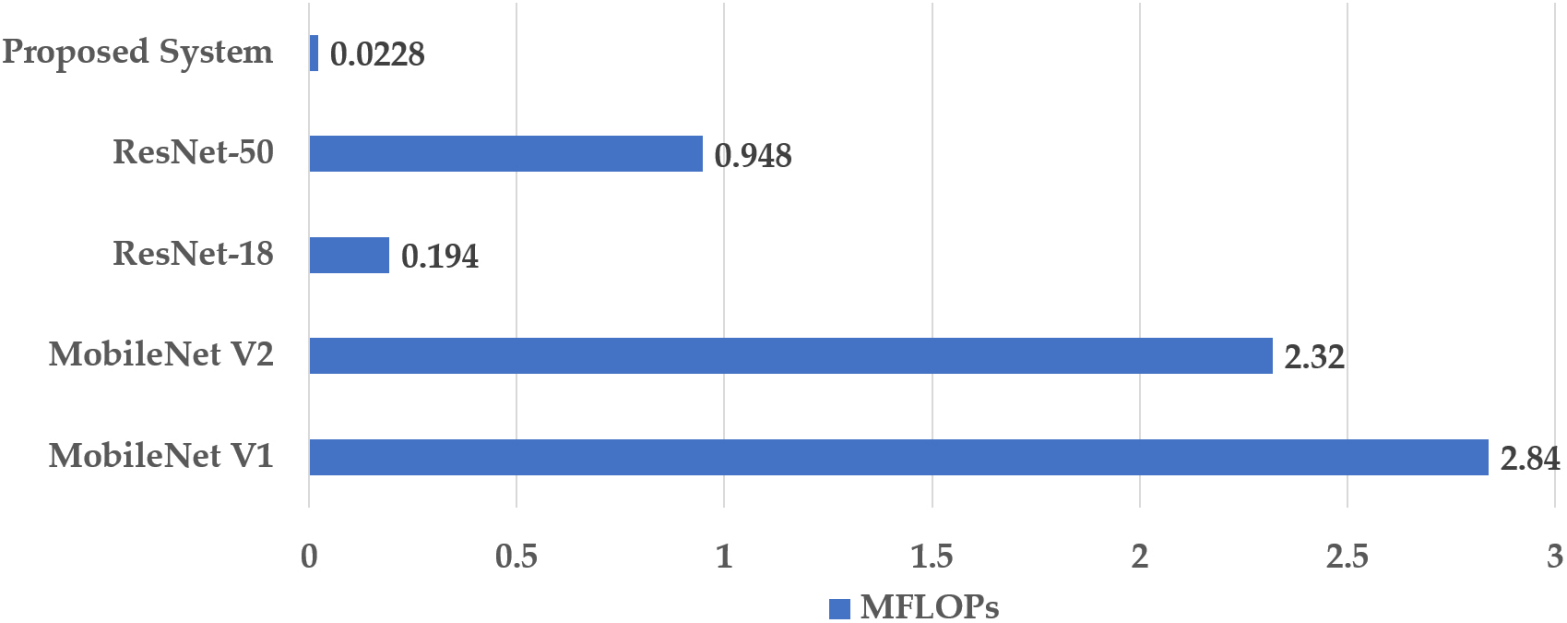
Comparative analysis based on MFLOPs.

Fig 22 presents a bar chart comparing the computational complexity of the proposed system with several state-of-the-art models, measured in MFLOPs.

The MFLOPs are computed for a single forward pass using the input image size of 32 × 32. The computational costs of MobileNet-V1 and MobileNet-V2 are the most significant, requiring 2.84 and 2.32 MFLOPs, respectively. The proposed approach has a minimal computational complexity of 0.0228 MFLOPs, indicating the reduction rate of around 99.2%, 99.0%, 97.7%, and 95.7% in comparison to MobileNet-V1, MobileNet-V2, ResNet-50, andResNet-18, respectively.

**Fig 23:**
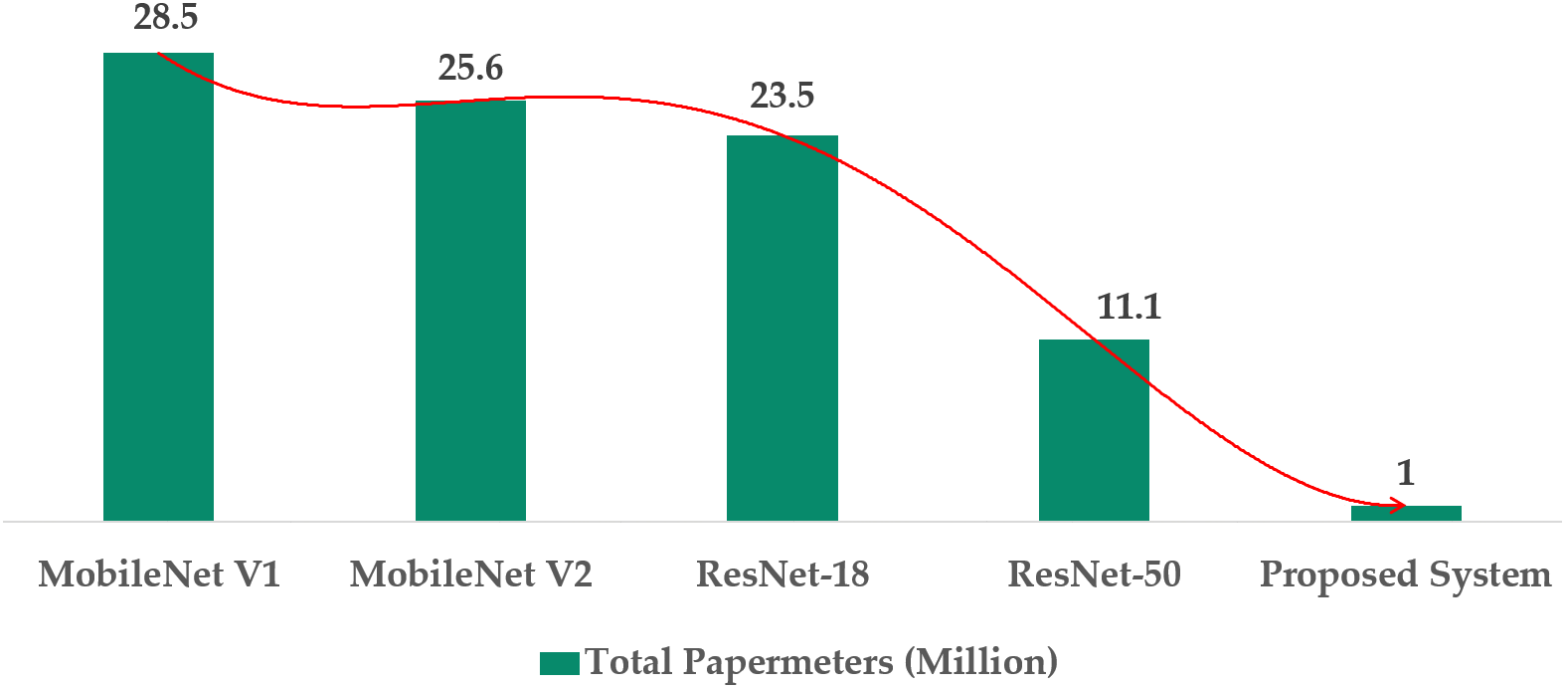
Comparative analysis based on total parameters.

Fig 23 presents the comparison of the total parameters (in millions) of various models. The suggested model contains 1 million parameters, notably fewer than the ResNet-18 (23.5 million) and ResNet-50 (11.1 million) models.

MobileNet V1 and MobileNet V2 have the parameter counts of 28.5 million and 25.6 million, respectively, resulting in a larger number of parameters than the proposed modified model. These findings demonstrate that the suggested method achieves an optimal balance between computational efficiency and resource consumption.

### 4.7 Comparative analysis of the proposed hybrid system with several developed systems

The performance analysis of the proposed model and other developed models on the defective wafer dataset [31] is given in Table 4. The proposed method outperforms the defect classification technique in [9] regarding complicated pattern detection, increasing the precision value by 1.83%.

**Table 4:** Comparison of the proposed hybrid approach with other methods based on average values of performance metrics.

Methods	Avg. Precision (%)	Avg. Recall (%)	Avg. F1-score (%)
[9]	93.81	93.79	93.76
[14]	93.30	89.10	90.71
Proposed method	95.64	93.34	94.42

The average F1-score and recall value of the proposed model notably improved by 3.71%,and 4.24% compared with the developed method in [14]. Table 5 compares performance metrics for defect classes among the proposed hybrid model, utilizing the wafers dataset [31], the developed method in [9], and [14].

**Table 5:** Comparison of the proposed hybrid approach with other systems based on the performance metrics.

Methods	Measure	Donut (%)	Edge-Ring (%)	Local(%)	Scratch(%)
[9]	Precision	94.19	98.04	85.11	91.40
	Recall	94.19	99.01	76.92	91.40
	F1-score	94.19	98.52	80.81	91.40
[14]	Precision	94.02	99.57	89.80	76.31
	Recall	93.88	99.32	81.91	50.40
	F1-score	93.91	99.44	85.53	60.17
Proposed method	Precision	95.12	99.71	91.59	85.51
	Recall	95.12	99.59	87.35	76.57
	F1-score	95.12	99.65	89.42	80.79

The proposed model achieved a 6.48% higher precision value, a 10.43% higher recall value, and an 8.61% higher F1-score value than the developed model in [9] for detecting ‘Local’ defective patterns. These results demonstrate that the proposed model outperforms the detection model in [9] in detecting small defective patterns within wafers. The proposed model outperforms the developed model in [14] by adeptly identifying intricate patterns and capturing long-range spatial relationships in the ‘Scratch’ and ‘Donut’ defect classes, boosting the precision values by 1.1% and 9.2%, respectively, and raising the F1-score to 95.12 and 80.79, respectively. Table 6 shows a comparative study on balanced and unbalanced datasets.

**Table 6:** Comparison of the proposed hybrid model and the base MobileNet model based on dataset balancing.

Models	Measure	Center (%)	Donut (%)	Edge-Local (%)	Edge-Ring (%)	Local (%)	Near-Full (%)	None (%)	Random (%)	Scratch (%)
Original MobileNet Model with the balanced dataset	Precision	94.15	94.12	90.13	97.88	76.72	97.37	97.11	93.73	70.22
	Recall	96.77	83.37	92.48	96.25	74.02	93.67	96.19	90.40	60.63
	F1-score	95.44	88.42	91.23	97.06	75.34	95.48	96.65	92.03	65.07
Proposed model with the imbalanced dataset	Precision	98.57	95.12	96.65	99.71	91.59	100	99.17	94.40	85.51
	Recall	99.57	95.12	95.45	99.59	87.35	99.00	90.73	96.72	76.57
	F1-score	99.07	95.12	96.04	99.65	89.42	94.74	99.45	95.55	80.79

The effectiveness of the suggested model with an imbalanced dataset [31] is examined through a comparison with the base MobileNet model trained on a balanced dataset that is constructed from the WM-811k dataset [31]. The proposed strategy boosted the recall values for the ‘Donut’, Near-Full, ‘Random’, and ‘Scratch’ faults, which represent a minority class in the dataset [31], in comparison to the baseline MobileNet model that utilizes balanced class distribution. In contrast to the baseline model, the recall metric increased by 11.75% for ‘Donut’, 5.33% for ‘Near Full’, and 6.32% for ‘Random ‘ defect classes. The proposed method achieved major improvements in identifying the minority class ‘Scratch’ by improving the recall value of 15.94% through the utilization of imbalanced defect classes. The results demonstrate that the proposed architecture overcomes the difficulties of class imbalance in the dataset by focusing on the minority defect classes and attains superior outcomes. The enhanced F1-score indicates that the hybrid model effectively equilibrates defect detection and reduces false positives. Table 7 compares the classification accuracy of the proposed model with another model [10].

**Table 7:** Comparison of the proposed hybrid approach with other developed methods- based on the classification accuracy of defect classes on the WM-811k dataset.

Methods	Defective Classes	Classification Accuracy (%)
[10]	Donut	92%
	Edge-Ring	99%
	Scratch	77%
	Local	80%
Proposed method	Donut	95.12
	Edge-Ring	99.65
	Scratch	97.34
	Local	86.54

The proposed model suppresses the developed model in [10] for capturing tiny defects within the wafers, improving classification accuracy by 6.54% in detecting ‘Local’ defect patterns. These outcomes demonstrate that using the wafer dataset [31], the proposed framework outperforms the wafer defect patterns recognition model in [10] in terms of identifying minor defects, complex patterns, and long-distance relationships of defects of the listed defect classes in this table.

**Fig 24:**
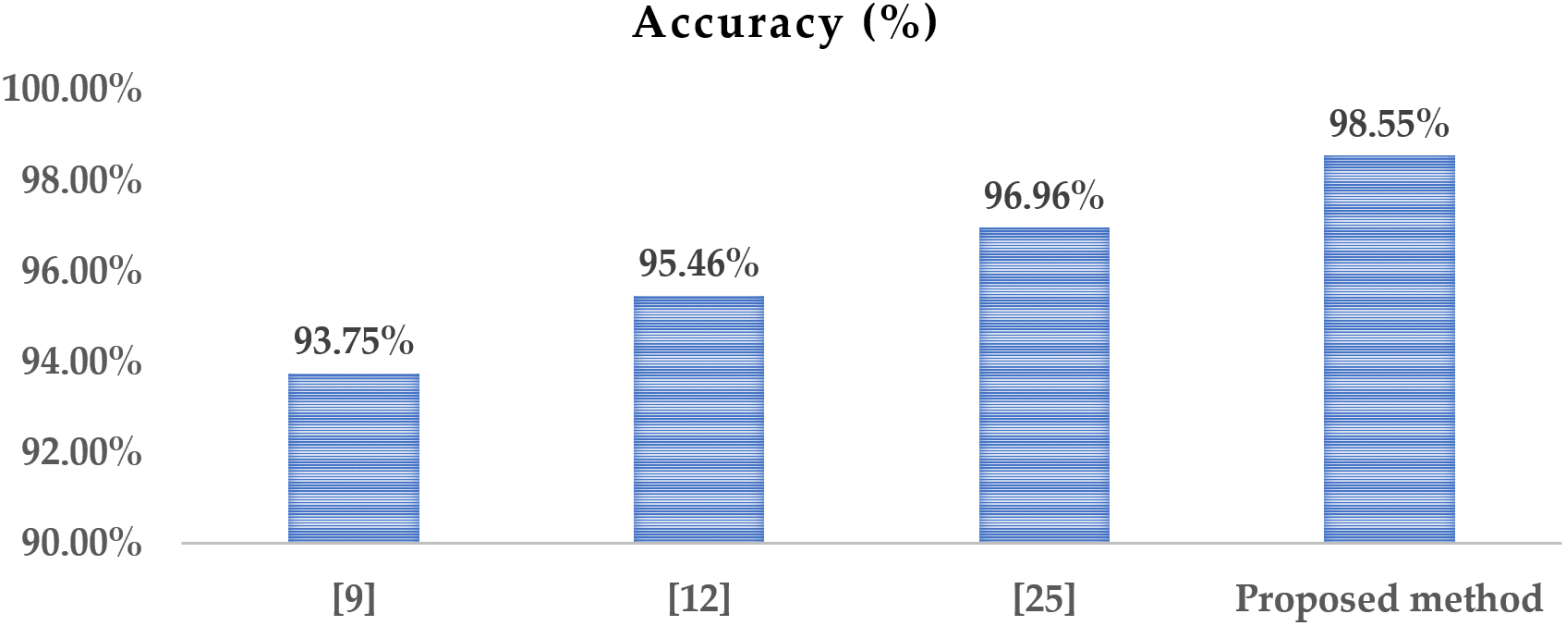
Comparison of the proposed hybrid approach with other methods based on accuracy.

Fig 24 illustrates the comparison among various categorization methods for defective wafers using the dataset [31].

The proposed method achieved accuracy levels 1.59% and 3.09% higher than those of the defect detection systems referenced in [25], which utilize the modified attention mechanism, and [12], respectively. The proposed hybrid strategy outperformed the technique established in [9], achieving a 4.8% improvement in accuracy.

## 5. Conclusions

Due to the numerous variances and sophisticated geometries of semiconductor defective wafer maps, noise and unpredictability in the production process make it difficult to identify small defects and complex patterns. Furthermore, as defects frequently interact across different spatial regions, it is critical to capture long-range dependencies inside defective wafers for accurate defect identification. This paper presents a modified MobileNet-based hybrid system to classify wafer defects using an ECOC-SVM classifier, which effectively addresses the identified issues. The suggested method uses the histogram equalization technique to enhance visibility in defective regions in wafers.

The swish function utilization in the proposed architecture significantly improves F1-score and AUC values to identify ‘Dount’ and ‘Scratch’, types of intricate wafer defect classes. The multi-head attention mechanism increases the modified model’s ability to identify long spatial relationships of defects in the ‘Edge-ring’, ‘Donut’, and ‘Scratch’ defect classes, resulting in boosted AUC and precision values. The severe class imbalance problem of the dataset WM-811k is mitigated by implementing the ECOC-SVM classifier model in the proposed architecture, resulting in notably raising the recall values for minority defect classes ‘Donut’, ‘Near full’, ‘Random’, and ‘Scratch’. The suggested method applies histogram equalization on defective wafer images to facilitate the accurate detection of small defects. This technique substantially improves the precision, recall, and AUC metrics for identifying tiny defects within the ‘Local’ defect class. The proposed hybrid model outperformed state-of-the-art models, such as MobileNet V1, MobileNet V2, ResNet-18, and ResNet-50, in terms of accuracy, learning parameters, and computational complexity. The proposed hybrid approach requires a minimal fraction of the MFLOPs compared to MobileNet V1 and MobileNet V2. The hybrid model attains a significant decrease in learning parameter count, with reductions of roughly 96.5% and 96.1% fewer parameters compared to MobileNet V1 and MobileNet V2, respectively. Various optimizers are used in the proposed hybrid model, among which the SGDM optimizer provides the best results. The proposed hybrid system with SGDM optimizer attains an accuracy of 98.55%, surpassing the accuracy of several defect pattern detection methods developed.

This performance study and comparative analysis proved that the hybrid system effectively addressed defective wafer map identification and improved accuracy in recognizing wafer defects. The proposed model outperforms the mentioned state-of-the-art models in classification accuracy, parameter size, and computational cost. Also, the ablation study in this paper provides evidence of the contribution of every major modification of the proposed hybrid approach. However, the proposed hybrid structure struggles to identify very thin ‘Scratch’ defects. Future research will focus on precisely recognizing the thin patterns within ‘Scratch’ type defective wafers by bridging the multi-head attention (MHA) and convolutional block attention module (CBAM).

## References

[1] Cortes C, Vapnik V. Support-vector networks. Mach Learn. 1995;20(3):273–97. doi: 10.1007/bf00994018

[2] Ma Y, Guo G. Support vector machines applications. Springer eBooks. 2014.

[3] Gunasekaran N, Zhai G, Yu Q. Sampled-data synchronization of delayed multi-agent networks and its application to coupled circuit. Neurocomputing. 2020;413:499–511. doi: 10.1016/j.neucom.2020.05.060

[4] Cover T, Hart P. Nearest neighbor pattern classification. IEEE Trans Inform Theory. 1967;13(1):21–7. doi: 10.1109/tit.1967.1053964

[5] Zhang S. Challenges in KNN Classification. IEEE Trans Knowl Data Eng. 2022;34(10):4663–75. doi: 10.1109/tkde.2021.3049250

[6] Shih DH, Yang CY, Wu TW, Shih MH. Investigating a machine learning approach to predicting white pixel defects in wafers—A case study of wafer fabrication plant F. Sensors. 2024;24(10):3144. 38793997 10.3390/s24103144 PMC11124928

[7] Mohiuddin M, Islam MS, Uddin J. Feature Optimization for Machine Learning Based Bearing Fault Classification. IJEEI. 2024;12(3). doi: 10.52549/ijeei.v12i3.5671

[8] Hoque MJ, Islam MdS, Uddin J, Samad MdA, De Abajo BS, Vargas DLR, et al. Incorporating Meteorological Data and Pesticide Information to Forecast Crop Yields Using Machine Learning. IEEE Access. 2024;12:47768–86. doi: 10.1109/access.2024.3383309

[9] Zheng H, Sherazi SWA, Son SH, Lee JY. A Deep Convolutional Neural Network-Based Multi-Class Image Classification for Automatic Wafer Map Failure Recognition in Semiconductor Manufacturing. Applied Sciences. 2021;11(20):9769. doi: 10.3390/app11209769

[10] Byun Y, Baek J-G. Pattern Classification for Small-Sized Defects Using Multi-Head CNN in Semiconductor Manufacturing. Int J Precis Eng Manuf. 2021;22(10):1681–91. doi: 10.1007/s12541-021-00566-2

[11] Misra S, Kim D, Kim J, Shin W, Kim C. A voting-based ensemble feature network for semiconductor wafer defect classification. Sci Rep. 2022;12(1):16254. doi: 10.1038/s41598-022-20630-9 36171470 PMC9519991

[12] Xu Q, Yu N, Essaf F. Improved Wafer Map Inspection Using Attention Mechanism and Cosine Normalization. Machines. 2022;10(2):146. doi: 10.3390/machines10020146

[13] Shin E, Yoo CD. Efficient convolutional neural networks for semiconductor wafer bin map classification. Sensors. 2023;23(4):1926. 36850523 10.3390/s23041926 PMC9960339

[14] Chen S, Zhang Y, Hou X, Shang Y, Yang P. Wafer map failure pattern recognition based on deep convolutional neural network. Expert Systems with Applications. 2022;209:118254. doi: 10.1016/j.eswa.2022.118254

[15] Jeon M, Yoo S, Kim S-W. A Contactless PCBA Defect Detection Method: Convolutional Neural Networks With Thermographic Images. IEEE Trans Compon, Packag Manufact Technol. 2022;12(3):489–501. doi: 10.1109/tcpmt.2022.3147319

[16] Sun X, Zhang B, Wang Y, Mai J, Wang Y, Tan J, et al. A Multiscale Attention Mechanism Super-Resolution Confocal Microscopy for Wafer Defect Detection. IEEE Trans Automat Sci Eng. 2025;22:1016–27. doi: 10.1109/tase.2024.3358693

[17] Limam K, Cheema S, Mouhoubi S, Freijedo FD. Deep Learning-Based Visual Recognition for Inline Defects in Production of Semiconductors. IEEE J Emerg Sel Top Ind Electron. 2024;5(1):203–11. doi: 10.1109/jestie.2023.3326092

[18] Shahroz M, Ali M, Tahir A, Gongora HF, Rios CU, Samad MA, et al. Hierarchical Attention Module-Based Hotspot Detection in Wafer Fabrication Using Convolutional Neural Network Model. IEEE Access. 2024.

[19] Deng G, Wang H. Efficient Mixed-Type Wafer Defect Pattern Recognition Based on Light-Weight Neural Network. Micromachines (Basel). 2024;15(7):836. doi: 10.3390/mi15070836 39064347 PMC11279086

[20] Hou X, Yi M, Chen S, Liu M, Zhu Z. Recognition and Classification of Mixed Defect Pattern Wafer Map Based on Multi-Path DCNN. IEEE Trans Semicond Manufact. 2024;37(3):316–28. doi: 10.1109/tsm.2024.3418520

[21] Ma C, Jie L, Yao Y, Xu T, Hu L. Spatial Attention Enhanced Wafer Defect Classification Algorithm for Tiny Defects. In2023 IEEE 15th International Conference on Advanced Infocomm Technology (ICAIT) 2023 Oct 13 (pp. 380-384). IEEE.

[22] López de la Rosa F, Gómez-Sirvent JL, Morales R, Sánchez-Reolid R, Fernández-Caballero A. Defect detection and classification on semiconductor wafers using two-stage geometric transformation-based data augmentation and SqueezeNet lightweight convolutional neural network. Computers & Industrial Engineering. 2023;183:109549. doi: 10.1016/j.cie.2023.109549

[23] Shim J, Kang S. Learning from single-defect wafer maps to classify mixed-defect wafer maps. Expert Systems with Applications. 2023;233:120923. doi: 10.1016/j.eswa.2023.120923

[24] Hossain SMM, Deb K, Dhar PK, Koshiba T. Plant Leaf Disease Recognition Using Depth-Wise Separable Convolution-Based Models. Symmetry. 2021;13(3):511. doi: 10.3390/sym13030511

[25] Chen S, Liu M, Hou X, Zhu Z, Huang Z, Wang T. Wafer map defect pattern detection method based on improved attention mechanism. Expert Systems with Applications. 2023;230:120544. doi: 10.1016/j.eswa.2023.120544

[26] Schlosser T, Friedrich M, Beuth F, Kowerko D. Improving automated visual fault inspection for semiconductor manufacturing using a hybrid multistage system of deep neural networks. J Intell Manuf. 2022;33(4):1099–123. doi: 10.1007/s10845-021-01906-9

[27] Tumpa PP, Islam MdS. Lightweight Parallel Convolutional Neural Network With SVM Classifier for Satellite Imagery Classification. IEEE Trans Artif Intell. 2024;5(11):5676–88. doi: 10.1109/tai.2024.3423813

[28] Howard AG, Zhu M, Chen B, Kalenichenko D, Wang W, Weyand T, Andreetto M, Adam H. Mobilenets: Efficient convolutional neural networks for mobile vision applications. arXiv preprint arXiv:1704.04861. 2017 Apr 17.

[29] Sandler M, Howard A, Zhu M, Zhmoginov A, Chen LC. Mobilenetv2: Inverted residuals and linear bottlenecks. In Proceedings of the IEEE conference on computer vision and pattern recognition 2018 (pp. 4510-4520).

[30] He K, Zhang X, Ren S, Sun J. Deep Residual Learning for Image Recognition. In: 2016 IEEE Conference on Computer Vision and Pattern Recognition (CVPR), 2016. 770–8. doi: 10.1109/cvpr.2016.90

[31] Wu M-J, Jang J-SR, Chen J-L. Wafer map failure pattern recognition and similarity ranking for large-scale data sets. IEEE Transactions on Semiconductor Manufacturing. 2015;28(1):1–12.

[32] Gonzalez RC, Woods RE. Digital image processing. 2nd ed. Upper Saddle River (NJ): Prentice Hall; 2002.

[33] Glorot X, Bordes A, Bengio Y. Deep sparse rectifier neural networks. In: Foster JA, Lutton E, Miller J, Ryan C, Tettamanzi AG, editors. Genetic programming. EuroGP 2002: proceedings of the 5th European Conference on Genetic Programming; 2002 Apr 3–5; Kinsale, Ireland. Berlin (Germany): Springer; 2002. p. 315–23.

[34] Ramachandran P, Zoph B, Le QV. Searching for activation functions [Preprint]. arXiv. 2017 Oct 17 [cited 2017 Oct 17]. Available from: https://arxiv.org/abs/1710.05941

[35] Vaswani A, Shazeer N, Parmar N, Uszkoreit J, Jones L, Gomez AN, et al. Attention is all you need. Advances in Neural Information Processing Systems. 2017;30.

[36] Dietterich TG, Bakiri G. Solving Multiclass Learning Problems via Error-Correcting Output Codes. jair. 1995;2:263–86. doi: 10.1613/jair.105

[37] Hastie T, Tibshirani R. Classification by pairwise coupling. Advances in neural information processing systems. 1997;10.

[38] Ahlawat S, Choudhary A, Nayyar A, Singh S, Yoon B. Improved Handwritten Digit Recognition Using Convolutional Neural Networks (CNN). Sensors (Basel). 2020;20(12):3344. doi: 10.3390/s20123344 32545702 PMC7349603

[39] Kurbiel T, Khaleghian S. Training of deep neural networks based on distance measures using RMSProp [Preprint]. arXiv. 2017 Aug [cited 2017 Aug]. Available from: https://arxiv.org/abs/1708.01911

[40] Kingma DP, Ba JL. ADAM: a method for stochastic optimization [Preprint]. arXiv. 2014 Dec [cited 2014 Dec]. Available from: https://arxiv.org/abs/1412.6980

